# Natural polymer based drug-loaded hydrogel platform for comprehensive care of acute spinal cord injury

**DOI:** 10.1016/j.mtbio.2025.101464

**Published:** 2025-01-07

**Authors:** Mingyu Zhang, Chunyu Xiang, Xin Zhen, Wenqi Luo, Xiaodong He, Fengshuo Guo, Renrui Niu, Wanguo Liu, Rui Gu

**Affiliations:** a Department of Orthopaedic Surgery, China-Japan Union Hospital of Jilin University, Changchun, 130033, People's Republic of China; b Department of Physical examination center, China-Japan Union Hospital of Jilin University, Changchun, 130033, People's Republic of China

**Keywords:** Injectable hydrogel, Integrated therapy, Dexamethasone, Spinal cord injury, Wound healing

## Abstract

Traumatic spinal cord injury typically occurs at significant depths and triggers rapid and severe physiological responses. It is commonly accompanied by oxidative stress disorders, lipid peroxidation, accumulation of toxic aldehydes, and edema among other symptoms. The management of this condition requires intricate surgical procedures and vigilance against postoperative complications. Slow wound healing is a major clinical challenge. In this study, we developed an injectable hydrogel-based smart drug delivery platform (OPDL gel) for the treatment of cord injuries and integrated postoperative wound care. The hydrogel encapsulates the glucocorticoid dexamethasone (Dex) through a borate ester bond and can respond to degradation caused by reactive oxygen species (ROS) and pH changes in the microenvironment of spinal cord injuries. The OPDL gel was injected into the lesion with a degradation period of 60 h, enabling a controlled and intelligent release of Dex. Additionally, poly-ε-lysine macromolecules within the gel can absorb toxic aldehydes present in the microenvironment via Schiff base reactions, thereby mitigating secondary progression of spinal cord injury. When locally applied to spinal cord injuries, the gel demonstrated good biocompatibility and had a protective effect on damaged neural structures. In addition, OPDL gel also exhibited excellent bactericidal properties, achieving a 100 % kill rate against microorganisms within 80 min and providing wound healing care comparable to a commercial product, Tegaderm™. Therefore, this multifunctional hydrogel drug-loading platform represents a novel approach for integrated treatment strategies in the clinical setting to address spinal cord injuries.

## Graphical abstract

**Figure:**
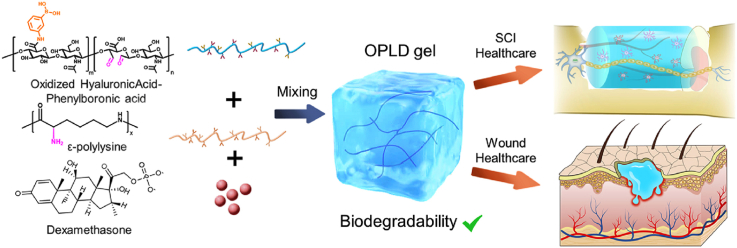

## 1 Introduction

Traumatic spinal cord injury (TSCI) is a common challenge in clinical practice [1]. The spinal cord is surrounded and protected by dense vertebral bones, making it vulnerable to acute injuries that are often accompanied by spinal damage. Fractured bone tissue can cause secondary harm to the spinal nerves and other organs [2,3]. Consequently, the condition of spinal cord injury remains highly unstable, with delayed medical intervention potentially resulting in paralysis or even life-threatening consequences [4]. Currently, the treatment options for acute spinal cord injury are limited [5]. Conventional approaches involve the surgical removal of foreign bodies and fractured bones from the wound site, along with the administration of high doses of glucocorticoids to suppress the inflammatory response caused by the injury [6,7]. Although glucocorticoids effectively inhibit inflammation at the site of spinal cord injury, they also supress immune responses in other parts of the body, such as mild postoperative incisional inflammation [8]. This inadvertently hampers surgical wound healing and increases susceptibility to bacterial invasion [9]. Patients undergoing postoperative steroid pulse therapy should also receive antibiotics to prevent bacterial infection at the wound site, which places an additional burden on organs such as the liver and kidneys [10,11]. Therefore, there is an urgent need for novel treatment strategies to address spinal cord nerve repair and postoperative wound care.

Spinal cord injuries (SCI) are typically deep with have large wounds, making it difficult to monitor the healing process and change dressings, similar to challenges faced with skin injuries [12,13]. Therefore, an optimal therapeutic strategy capable of delivering drugs to the injury site with minimal invasiveness while maintaining localized hormone levels represents a pivotal solution to this issue [14]. Hydrogels are highly water-absorbent biomaterials consisting of a cross-linked macromolecular network that exhibit structural resemblance to neural tissues and favorable biocompatibility [15]. These hydrogels possess the ability to fill cyst cavities and provide support for axonal growth and differentiation [16]. Certain hydrogel drug carriers can be administered via injection or implantation at the site of injury without eliciting additional immune responses [17]. These properties render hydrogels ideal delivery systems for drugs, growth factors, and stem cells by locally enriching them while mitigating the potential side effects associated with high-dose systemic treatments [18]. The use of dynamic chemical bonds is a typical strategy for fabricating injectable and self-healing hydrogel materials [19]. The injectability feature prevents any secondary damage resulting from surgical procedures, whereas the self-healing property enables adaptive coverage of irregular cavities and direct drug release into diseased tissues [20]. These properties theoretically fulfill the requirements for postoperative care of patients with SCI.

The human body is highly sensitive to foreign matter, and even trace amounts of chemical residues can cause severe side effects [21,22]. Consequently, biomedical materials used for deep injury treatment have exceptionally stringent requirements. Natural macromolecules generally possess superior biocompatibility [23,24]. Natural polycationic materials represent an emerging class of highly efficient antibacterial agents. Unlike antibiotics, which selectively target specific bacterial behaviors, polycationic materials exert their antimicrobial action by electrostatically interacting with the negatively charged outer membrane of bacteria and disrupting their structure, ultimately resulting in bacterial eradication [[25], [26], [27]]. This bactericidal mechanism significantly mitigates the development of bacterial resistance [28]. Therefore, the incorporation of antibacterial polycationic materials into hydrogel matrices offers a promising solution for preventing postoperative infections. Poly-ε-lysine (ε-PLL) is currently acknowledged as an antibacterial cationic polymer exhibiting excellent antimicrobial properties and biosafety profile [29]. Being composed of natural amino acids, this polymer can be enzymatically degraded after exerting its therapeutic effect within the body, making it an ideal substrate for the construction of antibacterial hydrogel drug carriers [[30], [31], [32]].

**Scheme 1:**
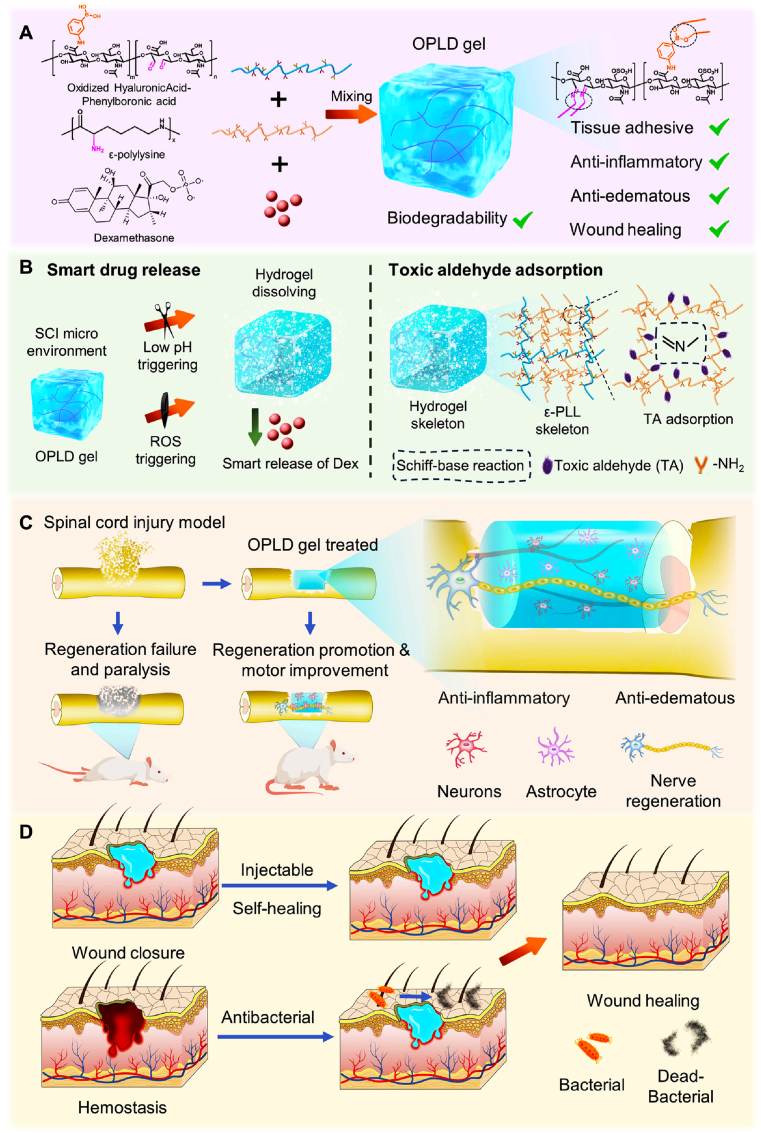
Schematic diagram of the fabrication of OPDL gel for promoting spinal cord injury healthcare. (A) Preparation and properties of OPDL gel. (B) Illustration of the intelligent drug release characteristics and toxic aldehyde adsorption of OPDL gel. (C) The mechanism of spinal cord injury healthcare using OPDL gel. (D) OPDL gel resists infection and promotes full thickness wound healing.

Herein, an injectable, drug-loaded, antibacterial hydrogel composed of natural polymers, abbreviated as OPLD Gel, was prepared for comprehensive treatment following spinal cord injury was prepared. As shown in Scheme 1A, OPLD gel consists of oxidized natural hyaluronic acid modified with phenylboronic acid (OHA-PBA) and poly-ε-lysine (ε-PLL), cross-linked by a Schiff base reaction. The glucocorticoid dexamethasone sodium phosphate (Dex) is incorporated into the gel through borate reactions and electrostatic interactions (Scheme S1), enabling intelligent release in response to both damaging reactive oxygen species (ROS) and a weakly acidic microenvironment. Additionally, the abundant amino groups in the OPDL gel can absorb toxic aldehydes present in the spinal cord injury (SCI) microenvironment to protect the spinal cord (Scheme 1B). The OPLD gel exhibited excellent injectability and self-healing properties, allowing its precise application at the site of spinal cord injury by using a syringe for effective care delivery (Scheme 1C). Furthermore, the OPLD gel demonstrated significant antibacterial activity, while effectively reducing the levels of harmful reactive aldehydes within the wound microenvironment. Importantly, when tested in animal models of acute spinal cord injury and skin infection, the application of OPLD gel significantly accelerated wound healing without any observable abnormalities in vital organs or hematological parameters (Scheme 1D).

## 2 Materials and methods

### 2.1 Materials

Sodium hyaluronate (HA, Mw = 400000–800000) was purchased from Shanghai Yuanye BioTechnology Co., Ltd. 3-Aminobenzeneboronic acid, N-hydroxysuccinimide (NHS) and 1-ethyl-3-(3-dimethylaminopropyl) carbodiimide (EDC·HCl), dexamethasone, were purchased from Aladdin Co. (Shanghai, China). The poly-ε-lysine (ε-PLL, Mw = 384.52, 95 %) was purchased from Energy Chemical, Shanghai Sane Chemical Technology Co., Ltd. Dulbecco's modified Eagle's medium (DMEM) was purchased from Gibco (USA). Methanol, sodium periodate, Purpald's reagent, and propionaldehyde were purchased from Aladdin Co. (Shanghai, China). Luria–Bertani (LB) agar broth and potato dextrose agar/broth were obtained from Hopebio, China. *S. aureus* (ATCC 6538) was obtained from the American Type Culture Collection (ATCC). Triton X-100, the CCK-8 cell viability test kit, and other biological reagent detection kits were purchased from Sevier Technology Co., Ltd. (Wuhan, China). All chemicals were of analytical reagent grade and were used without further purification.

### 2.2 Synthesis of the polymers

The HA (2 g) was dissolved in 200 mL of deionized water at 25 °C and the pH was adjusted to 5.8 using hydrochloric acid. Then, NHS (1.14 g), EDC·HCl (1.37 g), and 3-aminophenylboronic acid (1.34 g) were added to the solution. The mixture was stirred at 25 °C for 72 h. After dialysis in deionized water for another 3 d, the HA-PBA polymer was obtained by freeze-drying. The chemical composition of the polymer was determined by recording a ^1^H NMR spectrum (D_2_O) on a Bruker Fourier 300 instrument at 25 °C.

The OHA-PBA synthesis involved gradually adding 20 mL of a 0.65 mol/L sodium periodate solution dropwise into a 20 mL HA-PBA solution (0.1 g/mL). The resulting mixture was stirred in a dark environment, at room temperature (25 °C) for 6 h, and then quenched with a 15 mL ethylene glycol solution (50 % wt% in water). The product was purified using dialysis membranes (MW: 8–14 kDa) immersed in water. The solution was then lyophilized to obtain the final OHA-PBA product. The degree of oxidation (amount of introduced aldehyde groups) of OHA-PBA was determined using the Purpald reagent with a standard curve from propionaldehyde and hydroxylamine hydrochloride titration (Three times in duplicate and conducted five replicate tests on each sample). Briefly, the OHA-PBA sample (0.5 mg/mL) and Purpald reagent (10 mg/mL) were dissolved in a 1 M NaOH solution. The two solutions were mixed in equal volumes, incubated overnight at room temperature, and exposed to air. The absorption of the resulting purple solutions was measured by UV–vis spectroscopy at 550 nm (PerkinElmer Lambda-365). For the chemical structure of OHA-PBA, Fourier transformation infrared (FT-IR) spectra were recorded (Wave number range: 4000-400 cm^−1^; resolution: 4 cm^−1^; number of scans: 128 times) on a FT-IR spectrometry (INVENTRO-R, Bruker, Germany).

The grafting efficiency of 3-FPBA is determined by comparing the integrated areas of characteristic peaks on the benzene ring with methyl groups on HA backbone. When the grafting efficiency reaches 100 %, there should be a ratio of 4:3 between the integrated area of characteristic peaks attributed to 3-FPBA and methyl groups on HA backbone. In this work, for example, based on the integral ratio we obtained (0.27:3), we can deduce that 3-FPBA accounts for 0.067 %. Additionally, multiple tests were conducted to ensure result accuracy, and after statistical analysis, a final grafting rate of 6.7 % was obtained (with a standard deviation of 1.22).

### 2.3 Preparation and mechanical properties test of the OPLD gel

OHA-PBA (20 mg/mL), and ε-PLL (10 mg/mL) were dissolved separately in phosphate buffered saline (PBS, pH = 7.4), and the Dex was dissolved in DMSO (2 mg/mL). The OHA-PBA and ε-PLL solutions were then mixed at different volume ratio, with Dex solution dropped. After homogenizing, the OPDL gel was formed rapidly at 37 °C. A dynamic rheological analysis was performed using a rheometer (MCR302, Anton Paar). Rheological testing of the OPDL gel was conducted using a plate-plate geometry (25, 0.5 mm distance) and a dimethyl silicone trap to prevent drying. For the frequency sweep test, the frequency range was set to (0.1–100 rad/s) at a strain of 1 %. For the shear-thinning test, the shear rate range was set to (1–100 1/s). As for the self-healing performance test, OPDL gel was first subjected to a 1 % strain and maintained for 300 s, followed by applying an 800 % strain and maintaining it for another 300 s. This cycle was repeated three times with a final strain of 1 %. The interior structure of the hydrogel was examined using scanning electron microscopy (SEM; Merlin, Zeiss, Germany). For SEM testing, OPDL gel samples were initially pre-cooled in a temperature of 4 °C environment before being fractured in liquid nitrogen within a volume of 200 mL. The gel samples were freeze-dried for 24 h. After freeze drying, the Pd coating was applied to the OPDL gel samples before SEM testing at an acceleration voltage of 1 kV.

### 2.4 *In vitro* drug release performance testing

We utilized 24-well transwell plates with polycarbonate (PC) membranes having a pore size of 0.3 μm for in vitro microenvironment simulation. Initially, Dex was quantified using a standard UV spectroscopy curve by plotting its characteristic peak at 242 nm (R^2^ = 0.996). The 100 mg OPDL gel was injected into the chambers of a 24-well Transwell plate and left to settle until the bottom of the chamber pores was evenly filled. PBS (pH 6.5) was prepared by adjusting the pH using a standard solution of 0.01M hydrochloric acid, and the pH was determined using a PHS-2F INESA pH meter. The PBS buffer solutions (0.02M, pH∼6.5, and pH 7.4) were added to the outer chambers of the Transwell, and then the Transwell inner chamber with spread OPDL gel inside was placed in, ensuring that it was fully submerged. The PBS in the outer chambers of the transwell was replaced at different time intervals, and the amount of Dex released was determined using the UV standard curve method.

### 2.5 In vitro hydrogel degradation

The experiment utilized a 24-well transwell plate containing cell culture grade phosphate buffer solution (0.02 M, pH∼7.4 PBS) as the degradation medium. A volume of 1.5 mL of buffer solution was introduced into the lower chamber, while 200 μL of OPDL gel was injected into and immersed in the upper chamber prior to incubation at 37 °C. The entire culture medium was replaced daily and regular measurements were conducted on the gel mass until complete degradation occurred.

### 2.6 Biological safety testing of OPLD gel

PC12 and red blood cells were selected to conduct biosafety tests on the OPDL gels from three aspects: cell viability, cell migration, and cell proliferation. In briefly, PC12 cells were cultured in Dulbecco's Modified Eagle's medium (DMEM) supplemented with 10 % (v/v) fetal bovine serum (FBS) and 100 IU/mL penicillin, and streptomycin. Cells were seeded in 96-well plates at a density of 8000 cells/well. Cytotoxicity testing of the OPDL gel was performed using an extraction method. First, the sterilized OPDL gel prepared according to Fig. S3 (Gel-2) was added to sterile DMEM at different mass percentages. After incubating for 24 h, the DMEM medium samples containing the obtained OPDL gel were filtered through a 0.22-μm membrane to obtain the extract solutions. The extracted solutions were added directly to the plates. After incubation for an additional 24 h, the extract solutions were removed and the cells were washed three times with sterile PBS before being incubated with either fresh PBS solution containing a live/dead staining kit or fresh DMEM containing the CCK-8 reagent. Live/dead staining images were captured after half an hour, whereas absorbance measurements at 492 nm were taken after 1 h for the CCK-8 group. The cell viability (%) was calculated by normalizing the sample absorbance to that of the control wells (n = 5). For the cell proliferation experiment, all steps were the same except that the cell density per well was changed to 6000.

For the cell migration assay, PC12 cells (300,000 cells/well) were seeded in a 6-well plate. Subsequently, wounds were created at equal intervals in each well and washed three times with PBS. Pre-prepared OPDL gel media were added to the cell wells, followed by incubation at 37 °C. The wound coverage rate was determined as the ratio of the increased wound area to the initial wound area at different time points.

A commercial sheep red blood cell suspension was initially washed with PBS and prepared as a 4 % suspension of red blood cells (RBCs). Subsequently, OPDL gel was dispersed in physiological saline at various concentrations, with a 1 % Triton-100 solution serving as the positive control and a sterile 0.9 % sodium chloride solution as the negative control. Following centrifugation to collect 1 mL of RBCs, the supernatant was discarded, and the red blood cells were resuspended with an equal volume of sample solution before being incubated at 37 °C for 2 h. After centrifugation, the resulting supernatant was collected and the absorbance was measured at a wavelength of 540 nm. The hemolysis rate was defined as the percentage difference between the experimental and positive control groups after subtracting the background values.

### 2.7 In vivo spinal cord injury care experiment of OPLD gel

All animal testing procedures were approved by the Animal Ethics Committee of Jilin University. Female Sprague-Dawley rats, aged 6–8 weeks, were obtained from Liaoning Changsheng Biotechnology Ltd. and provided unrestricted access to food and water. Rats were anesthetized using intraperitoneal pentobarbital sodium at a dose of 5 mg/kg body weight. After disinfection, T10 laminectomy was performed to expose the spinal cord. In the sham group, only the spinal cord was exposed, whereas in the spinal cord injury (SCI) group, an injury model was created using a weightdrop device (C4P01-001; Shenzhen, China). A 40 g rod was dropped from a height of 50 mm onto the exposed dorsal surface of the spinal cord, causing an initial penetration depth of 2.5 mm. Following SCI induction, either a 10 μL solution of Dex (2 mg/mL), or a sterile 0.9 % sodium chloride solution as Blank, commercial 3M hydrogel Tegaderm™ named as Teg-gel, and OPDL gel were orthotopically injected using a microsyringe (26 G needle with inner diameter of 0.25 mm and outer diameter of 0.46 mm; Difa instrument Co., Ltd., Shanghai China). The rat urinary bladders were manually emptied twice daily until they regained autonomous urination ability. All animal experiments were conducted with 6 animals set as replicate samples in each experimental group.

### 2.8 In vitro antibacterial performance of OPDL gel

*E. coli* (ATCC 25922) and *S. aureus* (ATCC 6538) were selected for the antibacterial testing of the OPDL gel. All secondary strains were single colonies, purified, and incubated in the logarithmic phase before testing. The UV sterilized OPDL gel was pre-spread in a 48-well plate through a syringe, and 100 μL of bacterial suspension (10^8 CFU/mL) was then evenly dispersed on the surface of the OPDL gel by a coating rod. After co-incubating at 37 °C for different times (20, 40, 60, and 80 min), the surface of the OPDL gel was washed three times with 500 μL sterile saline to collect contacted bacteria, and then 50 μL of the washing solution was spread on the agar medium. After 12 h of incubation, the number of colonies was counted to evaluate the bactericidal properties.

### 2.9 *In vivo* full thickness wound healing performance of OPDL gel

Female mice weighing 35–40 g (Liaoning Changsheng Biotechnology, Ltd.) were used to create full-thickness wounds on their backs. The mice were randomly divided into different groups. Once the mice were anesthetized, their fur was removed and circular wounds with a diameter of 10 mm were made on the back of each mouse. The wounds were then treated with Tegaderm™, OPDL gel,S.aureus infected group (SA), or OPDL gel + SA, or left untreated. Photographs of the wound site were taken on Days 0, 3, 6, 9, and 12 of the healing process. ImageJ software was used to calculate the size of the wound areas in the photographs. All animal experiments were conducted with 6 animals set as replicate samples in each experimental group.

### 2.10 Statistical analysis

Data are presented as mean ± standard deviation (SD). Comparisons between two conditions were evaluated using an unpaired *t*-test. ns was not statistically significant. ∗P < 0.05, considered statistically significant. ∗∗P < 0.01 and ∗∗∗P < 0.001 were considered significant. Two-way ANOVA was conducted for comparison among multiple groups, followed by Hoa lm-Sidak analysis using EXCEL (Office 2016) and Origin 8.0.

## 3 Results and discussion

### 3.1 Preparation of the polymers and the OPLD gel

The OPLD gel is formed through direct crosslinking of benzeneboronic acid-modified HA and natural ε-PLL in an aqueous phase. First, 3-aminobenzeneboronic acid was grafted onto the main chain of HA via an EDC/NHS-catalyzed amidation reaction to obtain HA-PBA (Scheme S2). Subsequently, the adjacent dihydroxy groups in the HA-PBA segment were oxidized to active dialdehydes via periodic acid oxidation to obtain OHA-PBA (Scheme S3). The structure of HA-PBA was characterized by nuclear magnetic resonance spectroscopy. As depicted in Fig. S1, the triple proton peaks observed at δ = 7.5 are attributed to the phenyl ring on benzeneboronic acid. The grafting efficiency of 3-FPBA is determined by comparing the integrated areas of the characteristic peaks from the benzene ring with those of the methyl groups on the HA backbone, which is approximately 6.7 % ± 1.77. OHA-PBA was characterized using infrared spectroscopy, as shown in Fig. S2. Compared to the infrared spectrum of HA, a new absorption peak at 1753 cm^−1^ emerged, which can be attributed to the bending vibration of the active aldehyde carbonyl group, signifying successful oxidation. Additionally, the absorption peaks at 3300 cm^−1^ and 1581 cm^−1^ were assigned to amide vibrations, while the peaks at 1394 cm^−1^ and 1006 cm^−1^ were assigned to the in-plane bending vibrations of the hydroxyl groups on phenylboronic acid. These findings confirm the successful synthesis of OHA-PBA. Furthermore, we determined the degree of oxidation of OHA-PBA using the Purpald reagent method and found it to be 19.7 % ± 2.5 (Fig. S3C). The OPLD gel can rapidly form within a mere span of 10 s by directly mixing different concentrations of ε-PLL solution with an OHA-PBA solution, as illustrated in Fig. S3A. We calculated each functional group of OPDL gel through the following steps. Firstly, the molecular weight of the repeating unit (MRU) for HA was 379.32, and the degree of oxidation of OHA was tested to be about 19.7 %. Therefore, the MRU of OHA changed to 378.98. Then, after grafting with 3-FPBA (molecular weight was 136.94), the obtained OHAPBA was calculated with a grafting rate of 6.7 %, and the average MRU of OHA-PBA increased to 386.94. Finally, the average aldehyde equivalent was calculated to be 982.08 (0.5 × 386.94/0.197). The average amino equivalent was approximately equal to ε-PLL's MRU, which was calculated about 146.19, and the average Dex equivalent was approximately equal to Dex's MRU, which was calculated about 514.4. The optimal ratio selected in this manuscript is OHA-PBÃ10 wt%; ε- PLL∼5 wt%; Dex∼0.2 wt%, which can calculate an approximate molar ratio of active aldehyde groups, active amino groups, and Dex as being about 26:87:1. Therefore, it can be seen that the molar equivalent of active amino groups is much higher than that of active aldehyde groups and total molecular equivalent of Dex. The abundant presence of active amino groups in OPDL gel confers upon it both antibacterial properties and a high capacity for reactive aldehyde adsorption. From this observation, it can be concluded that there are more than sufficient remaining active amino equivalents in OPDL gel compared to both active aldehyde equivalents and total Dex molecules. Within a concentration range from 5 % wt., gels quickly form at multiple ratios between OHA-PBA and ε-PLL. However, after 2 h of standing, all gels except Gel-2 flowed down to the sample bottle (Fig. S3B). Therefore, we selected Gel-2 (OHA-PBÃ10 % wt., ε-PLL∼5 % wt.) as our model gel for subsequent testing.

### 3.2 Mechanical properties of the OPLD gel

**Fig. 1:**
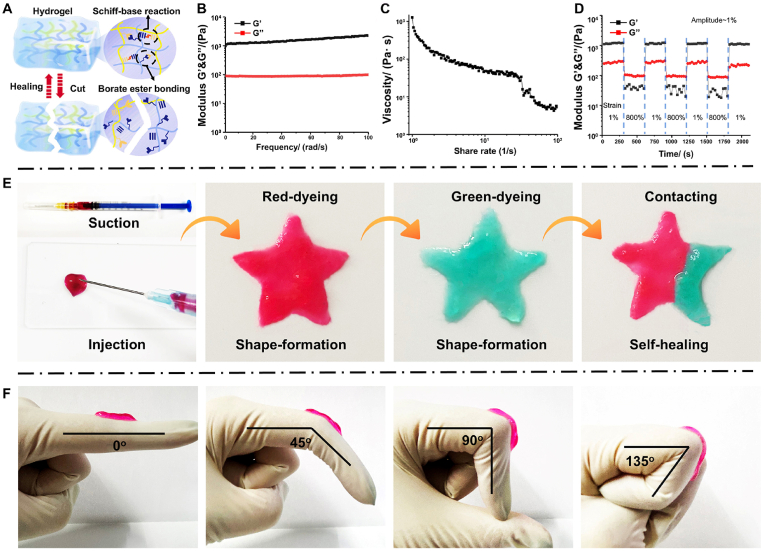
Characterization of the physical properties and rheological behavior of OPDL gel. (A) Illustration of the dynamic cross-linking mechanism of OPDL gel. (B) Rheological performance test of OPDL gel at a fixed amplitude of 1 %. (C) Viscosity change behavior of OPDL gel to increased shear rate. (D) Rheological performance analysis of OPDL gel under strain varying about 1 % and 800 % at a fixed time interval. (E) Evaluation demonstrates the "contact healing" behavior of OPDL gel, in which independent clumps form shapes with a certain tensile strength when in contact, allowing for further splicing and molding. (F) Photographs show the evaluation results of the bending conformity performance of OPDL gel on wrinkled joint surfaces.

Compared with cutaneous injuries, the treatment of deep tissue damage, particularly spinal cord injury, poses greater challenges [33]. Following surgical intervention, long-term IV administration is often necessary for drug delivery to the site of injury via the circulatory system [34]. This presents a significant obstacle to in situ drug delivery. The synthesis of cross-linked hydrogel networks via Schiff base formation (Fig. 1A) is an extensively investigated and validated approach for fabricating hydrogels with desirable attributes, including injectability, self-healing capability, and shape adaptability [35]. Rheological tests and macroscopic experiments were conducted to further investigate their characteristics (Fig. 1B). With increasing frequency, the OPDL gel exhibited a stable storage modulus (G′) > loss modulus (G’), indicating high elastic deformation. When subjected to shear strain at an increased rate, the viscosity of the OPDL gel decreased rapidly until it became almost fluid-like when the shear strain reached 100 rad/s, establishing a foundation for injectability (Fig. 1C). In addition to its injectability, the gel has remarkable self-healing properties owing to its dynamic chemical bonds. When administered to peripheral spinal cord injuries using a syringe, the gel exhibited adaptive behavior by gradually filling and conforming to the damaged area under the shear forces exerted by the surrounding tissues. As shown in Fig. 1D, subjected to alternating stress intervals of 1 % and 800 % for 300 s, the OPDL gel underwent a rapid transition between high elasticity and viscous flow. At an 800 % strain, the gel swiftly shifted from highly elastic deformation to viscous flow deformation with G'' > G′, indicating complete destruction of its crosslinked network. However, upon removal of the stress, G′ promptly returned to its initial state, demonstrating full reconstruction of the crosslinked network within the OPDL gel. Macroscopic results further validated these findings (Fig. 1E). The OPDL gel can be easily administered through a 1 mL medical syringe and molded according to the desired shape; upon contact, it rapidly heals into a homogeneous entity. Besides, in order to further validate the usability of OPDL gel in the scenarios, we conducted extrusion tests on OPDL gel using different syringe specifications, injection pressures, and extrusion times from a clinical perspective with recruited volunteers (Fig. S4). The results demonstrated successful extrusion of OPDL gel under all tested conditions. Additionally, rheological tests were performed on the OPDL gel before and after "contact-healing" to assess its healing efficacy. As shown in Fig. S5, it is evident that following the completion of "contact-healing," there was a slight reduction in the average storage modulus of the OPDL gel, with a calculated healing rate of approximately 92.3 % ± 1.37. Interestingly, this gel also exhibited notable adhesiveness and could withstand bending deformations ranging from 0° to 135° (Fig. 1F), which was attributed to electrostatic interactions between free amino cations within the gel and boronic ester bonds on OHA-PBA.

### 3.3 *In vitro* drug release performance testing of the OPLD gel

**Fig. 2:**
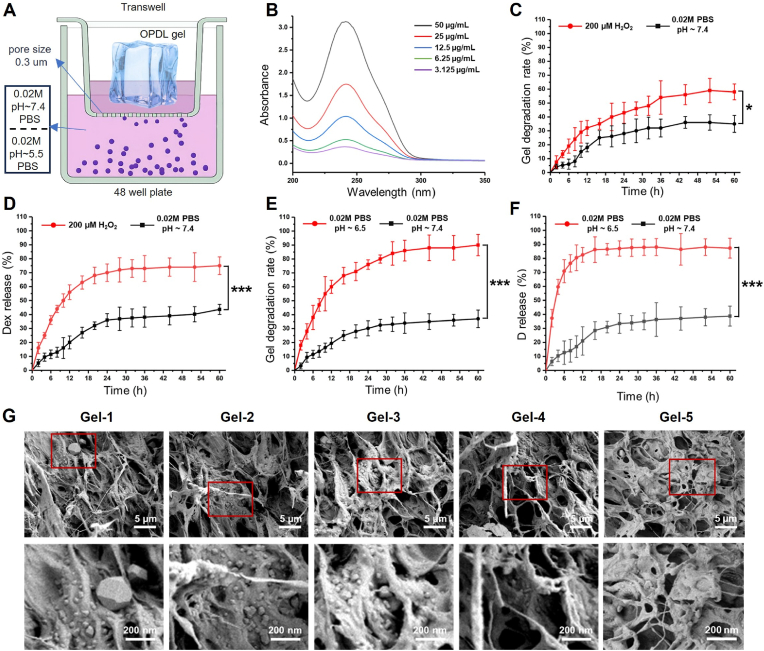
*In vitro* degradation and drug release behavior testing of OPDL gel. (A) Schematic diagram of in vitro degradation model for OPDL gel. (B) UV broad spectrum standard curve for the model drug Dex. (C) Degradation behavior of OPDL gel in H_2_O_2_ environment. (D) Drug release behavior of OPDL gel in H_2_O_2_ environment (E) Degradation behavior of OPDL gel in weak acidic environment of pH∼6.5. (F) Drug release behavior of OPDL gel in weak acidic environment of pH∼6.5. (G) Microscopic SEM photos of Dex in different gel sample deposition states.

To closely mimic the drug release behavior of OPLD gel within the microenvironment of spinal cord injury, we utilized 24-well transwell plates with pc membranes having a pore size of 0.3 μm and 0.02M pH∼7.4 PBS for in vitro microenvironment simulation (Fig. 2A). Initially, Dex was quantified using a UV spectroscopy standard curve by plotting its characteristic peak at 242 nm (R^2^ = 0.996) (Fig. 2B and Fig. S6A). As Dex is loaded into the OPDL gel through boronic ester bonds, which exhibit dual responsiveness to ROS and pH, we first simulated the degradation and release behavior of the OPDL gel in an ROS environment. As shown in Fig. 2C, after 60 h of incubation, the degradation rate of OPDL gel was only approximately 35.19 % ± 6.15 in weak alkaline body fluid (simulated using pH∼7.4 PBS at a concentration of 0.02M). However, in a highly ROS microenvironment (H_2_O_2_ 200 μM with 0.02M pH∼7.4 PBS as solvent), the degradation rate of the gel slightly increased, reaching a degradation rate approximately 57.5 % ± 5.74 within 60 h, which was 22.31 % higher than the 0.02 M pH∼7.4 PBS group. This is because ROS trigger the cleavage of boronic ester bonds, leading to the dissociation of the physical crosslinking points formed by phenylboronic acid and the hydrophobic drug Dex in the OPDL gel, ultimately resulting in an increase in the gel degradation rate. At the same time, there was a significant improvement in the release rate of model drug Dex in 200 μM H_2_O_2_ environment (Fig. 2D). Dex reached its release limit within 30 h, approximately at 75.5 % ± 6.34, while the control group (pH∼7.4 PBS at a concentration of 0.02M) only had a release rate of 43.65 % ± 3.65. Additionally, both the gel degradation rate and drug release rate were significantly enhanced in weakly acidic microenvironment (0.02M pH∼6.5 PBS) (Fig. 2E). Within 60 h, the gel degradation rate reached 87.3 % ± 7.11, nearly 2.25 times higher than that of PBS (0.02 M pH∼7.4 PBS) group degradation rate (38.76 % ± 7.13). The release rate of Dex is more sensitive in weakly acidic environments, reaching 86.26 % ± 9.16 in just 15 h, while the release rate is only 28.45 % ± 6.35 in weakly alkaline microenvironments (Fig. 2F). These results demonstrate that the OPDL gel can be triggered by both ROS and pH to sensitively release Dex. From the microscopic morphology of the OPDL gel (Fig. 2G), it can be observed that Dex was deposited uniformly in the gel network in different gel formulations. Further, we performed in vitro degradation experiments on the OPDL gel using medical-grade hyaluronidase injection (150 U/mL) and cell culture-grade pancreatic protease solution (0.25 mg/mL) as media. As depicted in Fig. S6B, the results demonstrated an accelerated degradation rate of the OPDL gel when exposed to hyaluronidase, reaching approximately 60 % after 60 h. However, the degradation of the OPDL gel exhibited limited sensitivity to pancreatic protease solution, with a degradation rate of only about 40 % after 60 h.

After a spinal cord injury, intense inflammatory reactions typically occur at the lesion site. However, these spontaneous immune system-mediated inflammatory responses often result in adverse effects such as swelling. If left uncontrolled, these side effects can cause further damage to the spinal cord and surrounding tissues, including compression of spinal nerves and local tissue death. Hormonal shock therapy refers to the immediate administration of high-dose corticosteroids following acute spinal cord injury in order to suppress the body's immune response and reduce the occurrence of these inflammatory side effects, thereby alleviating secondary damage formation. OPDL gel contains an adequate number of corticosteroids that can be directly injected into the injured site for sustained release within the traumatic microenvironment, maintaining the concentration of corticosteroids in this environment. These characteristics meet the requirements of hormone shock therapy for the treatment of deep cord injury cord injury treatment.

### 3.4 Biological safety testing of OPLD gel

**Fig. 3:**
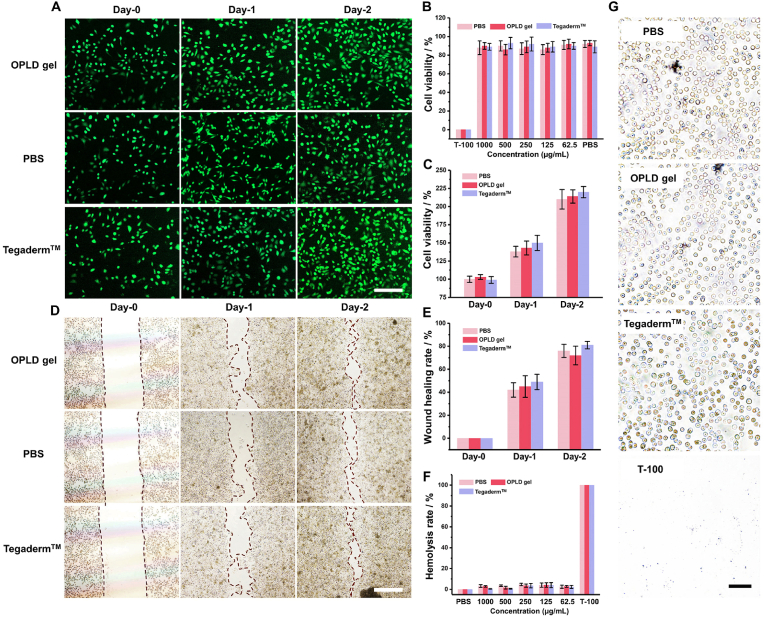
*In vitro* biosafety evaluation of OPDL gel. (A) Live/Dead staining results of PC12 cells after treatment with OPDL gel, PBS and commercial Tegaderm™ for different time periods. (B) Concentration-dependent toxicity test of OPDL gel on PC12 cells (C) Migration performance results of PC12 cells after treatment with OPDL gel, PBS and commercial Tegaderm™ for different time periods. Statistical analysis of (D) proliferation rate of PC12 cells after treatment with OPDL gel, PBS and commercial Tegaderm™ for different time periods (E) Statistical analysis of migration rate. (F) Blood cell compatibility test of OPDL gel, PBS and commercial Tegaderm™ (G) Micro-optical microscope photo of red blood cells after the test (scale bar was 100 μm). (For interpretation of the references to colour in this figure legend, the reader is referred to the Web version of this article.)

Good compatibility between cells and blood is essential for the development of medical gel carrier materials. To evaluate the viability, migratory ability, proliferative capacity, acute red blood cell lysis rate, and microscopic morphology of the OPDL gel, we selected the PC12 neuronal cell line and defibrinated sheep red blood cells as model cell strains. As depicted in Fig. 3B, even at a concentration of 1000 μg/mL, OPDL gel maintained a cell survival rate close to 90 %, which is comparable to that of the commercial gel Tegaderm™. Therefore, we chose the concentration of 1000 μg/mL for further testing purposes. As shown in Fig. 3A, during the 48 h incubation period, Ca-AM-stained PC12 cells exhibited bright green fluorescence, indicating the survival of healthy cells. There was a positive correlation between the incubation time and cell density. From the CCK-8 test, the statistical analysis revealed no significant differences between the OPDL gel and commercial gels or blank control groups (Fig. 3C). Moreover, the OPDL gel demonstrated an excellent migratory ability in PC12 cell migration tests. In vitro scratch experiments (Fig. 3D) revealed distinct boundaries of cellular wounds on day zero in all three groups. After co-incubation for one day, noticeable migration of PC12 cells occurred, and statistical analysis was conducted on the wound healing area after two days of co-incubation. As shown in Fig. 3E, the OPDL gel did not exhibit any statistically significant differences in cell migration performance compared to either the control group or Tegaderm™. However, a slight promoting effect on PC12 cell migration was observed with Tegaderm™. Furthermore, at the concentration of 1000 μg/mL, the OPDL gel demonstrated excellent affinity for blood cells (Fig. S7). As illustrated in Fig. 3F, within the co-incubation period of 2 h, the blood cell lysis rate in the OPDL gel group remained below 5 %. Fluorescence microscopy also revealed that red blood cells exhibited a typical concave disc shape (Fig. 3G), indicating that the OPDL gel did not induce non-lytic adverse effects in red blood cells. Furthermore, in order to enhance the accuracy of the experimental findings, we specifically selected primary neuronal cells to assess the relevant biosafety indicators of OPDL gel. As depicted in Fig. S8, our results demonstrated that OPDL gel exhibited favorable biocompatibility with primary neuronal cells, consistent with the observations made with PC12 cells. Additionally, we evaluated the impact of OPDL gel on cell migration performance using a similar methodology as described in this manuscript. Notably, no promotion of migration or differentiation was observed in primary neuronal cells treated with OPDL gel. Overall, the biocompatibility of the OPDL gel with both nerve and blood cells was comparable to that of the commercially available wound-healing gel Tegaderm™. These findings provide a solid foundation for the future in vivo applications of OPDL gels.

### 3.5 *In vivo* spinal cord injury care experiment of OPLD gel

**Fig. 4:**
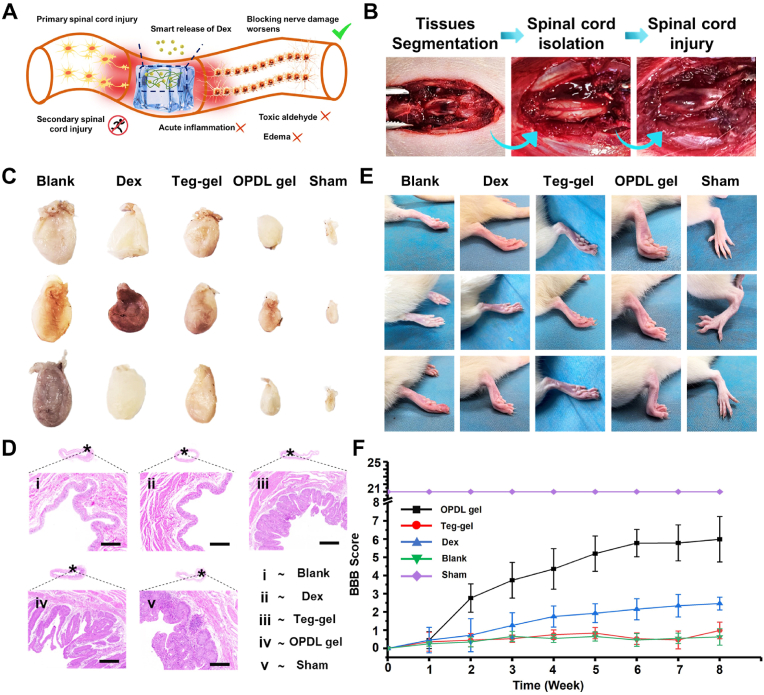
OPDL gel improved the behavioral function and pathology of SCI rats after treatment. (A) Schematic diagram of OPDL gel for reducing secondary injury after SCI by intelligent controlled release of Dex and adsorption of active aldehydes. (B) SCI animal model establishment process and real-time wound photos. (C) Comparative photos of bladder volume of SCI rats after treatment with different experimental samples. (D) HE pathological sections of bladder of SCI rats after the experiment. (E) Photos of hind limb behavior of SCI rats in different experimental groups after treatment. (F) BBB score index of motor ability of SCI rats after the experiment. Each animal experiment is set with 6 parallel samples.

A severe inflammatory response was observed after acute SCI (Fig. 4A). ROS levels increase rapidly in the injured microenvironment, leading to the recruitment of phagocytes for the clearance of apoptotic cells and necrotic tissues. This subsequently induces local edema and elevates toxic aldehyde content, resulting in secondary trauma to the spinal cord. However, Dex, the glucocorticoid present in the OPDL gel, exhibits significant potential to delay edema onset and decelerate secondary spinal cord injury while promoting its repair. Consequently, we established a stepwise SD rat model of spinal cord injury (Fig. 4B). As an excretory organ in mammals, the bladder is often affected by central nervous system damage following spinal cord injury, leading to lower limb dysfunction and impaired urinary function. The incomplete emptying of urine from the bladder causes urine retention, which eventually results in bladder enlargement. Following treatment with the different experimental groups, rat bladder tissues were extracted for comparative analysis. As illustrated in Fig. 4C, upon completion of the treatment period, rats from the control group exhibited substantial volume enlargement of their bladders, reaching up to 422 % compared to sham group volume. The Dex treated group and Teg-gel treated groups exhibited similar bladder volumes, approximately 339 % compared to the sham group volume, suggesting limited protective effects of Dex and Tegaderm™ on urinary nerve recovery during spinal cord injury. However, rats treated with OPDL gel demonstrated remarkable outcomes, with only a slight increase, about (109 %) in bladder volume after treatment; some rats even partially regained autonomous urination function. Additionally, a pathological analysis was conducted on animal bladders, as depicted in Fig. 4D. All experimental groups displayed noticeable inflammation compared to the sham group, as shown in Fig. S9, the zoomed figures showed that the OPDL gel group exhibited significantly milder inflammatory indicators than the other groups. The bladder tissue thickness decreased in the blank group (Fig. S10), accompanied by loose cell arrangement and localized areas of slight bleeding, indicating edema. Conversely, the bladders of the OPDL gel group resembled those of the blank control group, with well-preserved morphology and tissue integrity despite some focal inflammatory reactions. Furthermore, locomotor behavior score in rats serves as another crucial indicator for assessing the level of spinal cord injury recovery. As depicted in Fig. 4E, rats in the blank group exhibited no lower-limb motor ability after treatment completion and were unable to achieve joint flexion; however, significant motor ability recovery was observed in the OPDL gel group, with some rats even achieving immediate hindlimb support. The Dex and Teg-gel groups demonstrated poorer recovery, achieving only slight bending of the lower limb joints. BBB scores aligned with the rat locomotor behavior (Fig. 4F), wherein the OPDL gel group scored significantly higher than the other experimental groups. Although Dex also contributed to post-injury recovery to some extent, its effect was inferior to that of the OPDL gel.

Gait analysis tests were performed on experimental animals. After an 8-week treatment period, stride length, print area, step sequence, and stance width were calculated to assess motor ability. As shown in Fig. S11, there was no significant difference in the stride length of the forelimbs between the different groups; however, compared with the forelimbs, there was a noticeable decrease in the stride length of the hind limbs. This is due to spinal cord injury affecting rats' locomotor ability, with some individuals completely losing motor function and relying solely on dragging for movement. The Blank and Teg-gel groups did not exhibit significant therapeutic effects. Although there was a slight improvement in hind limb motor ability after Dex treatment, it was not statistically significant, whereas the OPDL gel treatment group showed substantial improvement and demonstrated statistically significant differences when compared to other experimental groups. Other indicators further supported this conclusion: paw area in the OPDL gel treatment group was significantly higher than that of other groups, and its step sequence accuracy had evident recovery effects compared to other experimental groups; however, although there was an increase in support width after OPDL gel treatment for hind limbs, it did not reach statistical significance. These results demonstrate that, compared to blank Dex, OPDL gel exhibited more pronounced effects on spinal cord injuries.

### 3.6 Mechanism and pathological analysis of OPDL gel in the treatment of spinal cord injury

**Fig. 5:**
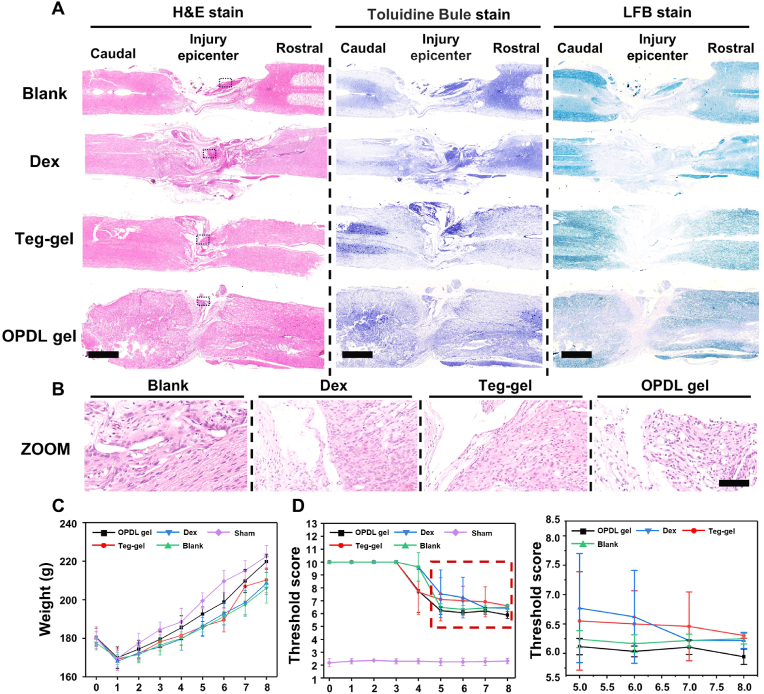
(A) H&E staining, TB staining and LFB staining images of spinal cord tissues from experimental rats. Scale bar = 500 μm. (B) Zoomed views of the typical area in the H&E stain slices. Scale bar = 50 μm. (C) Body weight change curve of experimental SCI rats. (D) Pain reflex score of rats using the Aesthesio® Von Frey Filaments method. Each animal experiment is set with 6 parallel samples.

To further evaluate the therapeutic efficacy of SCI, a comprehensive histological analysis of the experimental animal spinal cord was conducted using pathological section staining after the 8-week treatment period. As shown in Fig. 5A, the animals in the blank group treated with PBS exhibited defects and minimal signs of healing at the injury site. The free Dex and Teg-gel groups showed partial recovery; however, they displayed limited tissue continuity and low cell density within the spinal cord. Conversely, the OPDL gel treatment group exhibited superior recovery outcomes, with noticeable healing observed at the center of the injury despite exceeding 50 % loss of spinal cord integrity. Furthermore, residual inflammatory reactions were observed at all injury sites in the experimental groups after eight weeks of treatment; nevertheless, the inflammatory cell density was relatively lower in the OPDL gel treatment group (Fig. 5B).

Neurofilaments, also known as Nissl bodies, are alkaline granules or clumps unique to neurons that accumulate in the cell body and dendritic base [36]. These serve as markers of neuronal loss and damage. By observing the content of Nissl bodies in the local area of spinal cord injury samples using Toluidine Blue (TB) staining (Fig. 5A), it was observed that both Dex and Teg-gel treatments resulted in an increase in Nissl body content compared to the blank control group. However, the OPDL gel group exhibited a more significant preservation of Nissl bodies in the SCI tissue. This indicates that OPDL gel plays a beneficial role in protecting spinal nerves after spinal cord injury by facilitating the sustained release of glucocorticoids, thereby delaying edema occurrence post-injury and reducing secondary spinal cord injury severity. In addition to Nissl bodies, demyelination is a representative indicator of nerve damage. Myelin sheath damage can cause conduction blockage and induce paralysis; therefore, Luxol Fast Blue (LFB) staining was used to label the spinal cord samples (Fig. 5A). The OPDL gel-treated group exhibited a significant reduction in demyelination compared to the other experimental groups. Interestingly, there were no notable differences in demyelination between the Dex, Teg-gel, and blank groups. This implies that only the OPDL gel provided protection to neural tissue following spinal cord injury, whereas the other samples failed to alleviate secondary damage. Furthermore, the macroscopic vital signs of the experimental animals were consistently correlated with the pathological analysis findings (Fig. 5C). Rats treated with OPDL gel demonstrated healthier weight gain over time, significantly surpassing the other experimental groups. The lower limb tactile reflex after spinal cord injury served as an important indicator for assessing recovery progress (Fig. 5D). Von Frey Filaments were used to measure the minimum force required to stimulate lower limb movement speed. The results indicated that all experimental animals began exhibiting responses to stimulation from week three onwards. The OPDL gel group displayed superior recovery effects and showed the fastest recuperation period during weeks 4–5 before partially regaining reflex ability at the end of 8 weeks of treatment. These findings suggest that the application of OPDL gel after SCI has beneficial effects on neural cell protection and damaged spinal cord repair.

**Fig. 6:**
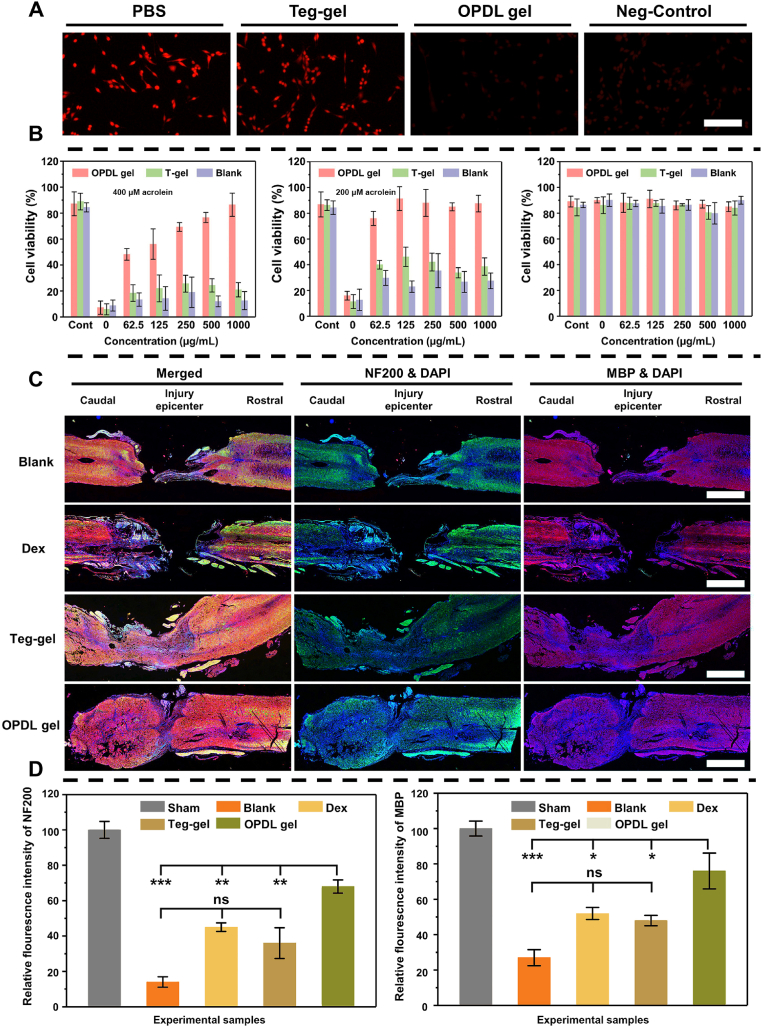
OPDL gel reduces the content of toxic aldehydes in the spinal cord microenvironment after SCI, protects the spinal cord and neurons, and reduces the degree of secondary injury. (A) Fluorescence fiber microscope images of PC12 cells stained with AcroleinRED after treatment with OPDL gel and Tegaderm™ (named Teg-gel in the Figures) in an environment with 200 μmol L^−1^ acrolein. (B) Viabilities of PC12 cells after incubation with OPDL gel for 24 h under different environments contain concentrations of 400 μmol L−1200 μmol L−1 acrolein-induced and PBS respectively. (C) Representative immunohistochemical staining images of the animal tissues, DAPI (nuclei, blue), MBP (axon, red) and NF200 (neuron, green). Scale bar = 500 μm (D) Statistical and quantitative analysis of the relevant indicators. (For interpretation of the references to colour in this figure legend, the reader is referred to the Web version of this article.)

The SCI microenvironment is susceptible to excessive accumulation of ROS and nitrogen species (RONS), which attack unsaturated fatty acids in situ, leading to oxidative reactions and the production of highly toxic endogenous aldehydes such as acrolein. Excessive levels of toxic aldehydes can cross-link protein molecules rich in amino groups and nucleic acids, thereby exacerbating secondary damage [37]. Therefore, reducing the content of toxic aldehydes in the SCI microenvironment of spinal cord injury can effectively protect the spinal cord tissue. The ε-PLL segment in OPDL gel contains a large number of amino groups that strongly bind to toxic aldehydes. Thus, we conducted an in vitro test on the adsorption capacity of the OPDL gel for toxic aldehydes. As shown in Fig. 6A, using the acrolein red probe as an indicator, PC12 cells cultured with PBS and Teg-gel exhibited significant red fluorescence, indicating erosion by toxic aldehydes; however, almost no fluorescence was observed in the OPDL gel or negative control groups. Besides, the levels of aldehydes in PC12 cells before and after exposure to the gel were characterized. As shown in Fig. S12, the quantification results of toxic aldehydes are consistent with microscopic images. The presence of OPDL gel significantly reduces toxic aldehyde levels in PC12 cells compared to both the PBS group and Teg-gel group, with levels almost similar to those observed in the blank group. This indicates that experimental cells were not affected by toxic aldehydes present in their microenvironment. Additionally, as depicted in Fig. 6B, the cell viability experiment quantitatively evaluated the pronounced neuronal cell death upon exposure to a concentration of 400 μmol/L acrolein within the microenvironment. Even at half the concentration (200 μmol/L), the cell survival rate remained below 50 %. However, when the OPDL gel was introduced, a noticeable enhancement in the cell survival rate was observed, demonstrating a concentration-dependent protective effect. Moreover, both the OPDL gel and Teg-gel exhibited favorable compatibility with PC12 cells in the absence of cytotoxic aldehydes.

Neurofilament-200 (NF-200) serves as a prominent indicator of nerve fiber regeneration, whereas myelin basic protein (MBP) is an alkaline protein that is highly enriched in nerve cells. Fluorescence labeling was performed to assess the extent of remyelination at the SCI site of spinal cord injury using NF200 and MBP. As depicted in Fig. 6C, the group treated with OPDL gel exhibited a higher abundance of neurons and increased myelin density at the injury site compared with the other groups. Both Dex and blank control groups were unable to maintain spinal cord integrity in the same section because of severe damage. Quantitative analysis of both fluorescent markers at the injury site revealed that OPDL gel treatment resulted in significantly higher axonal density (NF200∼72.3 %, MBP∼78.9 %) compared to other experimental groups and control group (NF200∼11.8 %, MBP∼23.6 %). Although Dex and Teg-gel also demonstrated some promoting effects on spinal cord injury, their therapeutic efficacy was far inferior to that of the OPDL gel, owing to Dex's intelligent release mechanism and its adsorption capacity for toxic aldehydes. These experimental results indicate that the OPDL gel exhibits favorable protective effects on neuronal cells and myelin structure after spinal cord injury, thus holding the potential for clinical application.

### 3.7 *In vivo* full thickness wound healing performance of OPDL gel

**Fig. 7:**
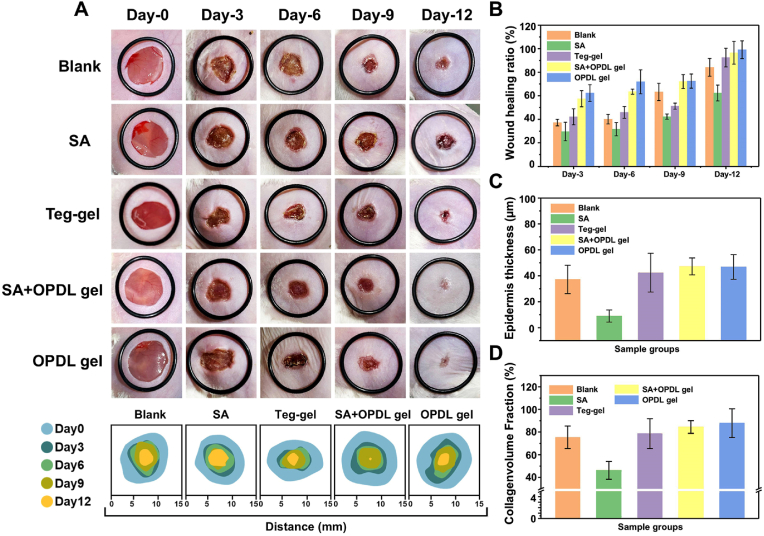
OPDL gel accelerates wound healing of full thickness wound infection in vivo. (A) Representative images of skin wound healing treated with OPDL gel, OPDL gel + SA, Tegaderm™, SA, and PBS (n = 6) at different time points. (B) Statistical analysis of the degree of skin wound healing in experimental animals at different time points. Statistical analysis of (C) dermal thickness and (D) collagen filling after skin healing in experimental groups. Each animal experiment is set with 6 parallel samples.

**Fig. 8:**
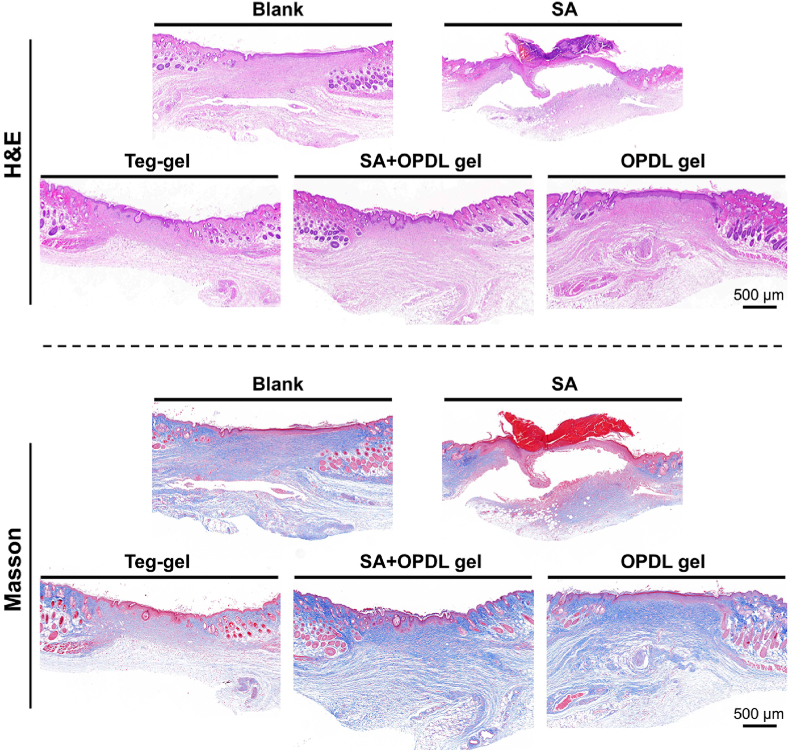
HE-stained sections of wound healing local tissues of animals in different experimental groups after treatment and digital scans of Masson-stained sections. Scale bar = 500 μm.

Traumatic spinal cord injury or spinal cord injury surgery often leads to the development of full-thickness skin wounds. Postoperative wound care can effectively reduce the incidence of infection, alleviate the organ metabolic burden, and mitigate patient suffering. The OPDL gel with ε-PLL segments possesses abundant amino groups, resulting in a highly positive surface charge that efficiently eradicates bacteria. An *in-vitro* contact antibacterial test was conducted on the OPDL gel. As shown in Fig. S13, the OPDL gel demonstrated remarkable contact killing activity against both *S. aureus* (Fig. S13A) and *E. coli* (Fig. S13B), achieving complete eradication of the bacteria at a concentration level of 10^7 CFU/mL within 80 min. This finding substantiates the antimicrobial properties of OPDL gel as a medical-grade hydrogel. The wound-healing efficacy of the OPDL gel was evaluated using a mouse model experiment involving full-thickness skin trauma. From the perspective of wound healing rate, as illustrated in Fig. 7A, the group treated with OPDL gel exhibited the highest rate of skin healing within 12 days, even demonstrating an 11 % improvement compared with commercial gel Tegaderm™ (Fig. 7B). In the *Staphylococcus aureus* (SA) infection group, evident purulent tissue was observed at the wound site until day 6, when the infection was preliminarily controlled and entered the healing process. However, animals in the experimental group treated with OPDL gel exhibited a favorable trend towards healing, even when infected with SA. To further investigate the effects of wound healing, skin tissues from the wounds were collected after completion of the experiments and subjected to hematoxylin and eosin/Masson staining for pathological analysis. The results of HE staining, as depicted in Fig. 8, demonstrated that although the SA-infected group initiated the healing process, a central wound defect persisted. Conversely, complete initial healing with granulation tissue completely filling the skin defects was achieved in all the other experimental groups. This was particularly evident in the OPDL gel-treated group, in which functional organelles and pores emerged. Notably, animals treated with OPDL gel exhibited the greatest dermal thickness compared to the other experimental groups (Fig. 7C). Masson's section results (Fig. 7D) revealed that collagen filling was most pronounced in the wound tissue of the OPDL gel treatment group. At this stage, remnants of partially detached scabs were observed in the blank control group, whereas skin integrity remained compromised in the SA-infected group. Furthermore, wounds treated with OPDL gel displayed elevated levels of collagen filling and scar tissue maturity comparable to Tegaderm™. These findings substantiate that OPDL gel provides valuable support for full-thickness postoperative wound healing, and its efficacy slightly surpasses that of commercially available Tegaderm™ gels.

## 4 Conclusion

This study presents a responsive injectable hydrogel platform loaded with Dex for comprehensive treatment of SCI. The dynamic hydrogel drug carrier was prepared through a simple mixing process involving Schiff base reaction between OHA-PBA and ε-PLL, as well as boronic ester reaction. The OPDL gel exhibited excellent self-healing and injectability properties, effectively encapsulating the glucocorticoid Dex via boronic ester bonds and hydrogen bonds and intelligently releasing it in response to excessive pH and ROS levels in the SCI microenvironment. This mechanism effectively alleviates secondary injuries, such as edema, following acute injury. Additionally, the high-density amino groups from ε-PLL within the OPDL gel efficiently adsorbed toxic aldehyde molecules in the microenvironment, providing significant protection for PC12 cells. In situ injection of the OPDL gel significantly accelerated the SCI healing process. Furthermore, the OPDL gel not only promoted full thickness wound healing, but also effectively prevented wound infection. Therefore, OPDL gel exhibits tremendous potential as an integrated material for spinal cord injury care.

## CRediT authorship contribution statement

**Mingyu Zhang:** Writing – review & editing, Writing – original draft, Methodology, Investigation, Data curation. **Chunyu Xiang:** Methodology, Formal analysis, Data curation. **Xin Zhen:** Software, Formal analysis, Data curation. **Wenqi Luo:** Supervision, Formal analysis, Data curation. **Xiaodong He:** Methodology. **Fengshuo Guo:** Data curation. **Renrui Niu:** Software, Methodology. **Wanguo Liu:** Writing – review & editing, Writing – original draft, Resources, Funding acquisition. **Rui Gu:** Writing – review & editing, Supervision, Resources, Funding acquisition.

## Declaration of competing interest

The authors declare that they have no known competing financial interests or personal relationships that could have appeared to influence the work reported in this paper.

## Data Availability

Data will be made available on request.
