# Supplementary material for: Natural polymer based drug-loaded hydrogel platform for comprehensive care of acute spinal cord injury
Source: Mater Today Bio. 2025 Jan 7;31:101464. doi: 10.1016/j.mtbio.2025.101464 (PMC11783013; doi:10.1016/j.mtbio.2025.101464)
Supplement: Multimedia component 1 [file mmc1.docx]

**Supporting Information**

Natural polymer based drug-loaded hydrogel platform for comprehensive care of acute spinal cord injury

Mingyu Zhang^a^, Chunyu Xiang^a^, Xin Zhen^b^, Wenqi Luo^a^, Xiaodong He^a^, Fengshuo Guo^a^, Renrui Niu^a^, Wanguo Liu^a^*, Rui Gu ^a^*

^a^ Department of Orthopaedic Surgery, China-Japan Union Hospital of Jilin University, Changchun, 130033, People's Republic of China

^b^Department of Physical examination center, China-Japan Union Hospital of Jilin University, Changchun, 130033, People's Republic of China

^*^ Corresponding author.

E-mail addresses: WL: liuwanguo6016@jlu.edu.cn, RG: gurui@jlu.edu.cn;

**Content:**

**Figures and schemes.**

***Scheme S1****.* Schematic diagram of the loading mechanism of OPDL gel for Dex molecules.

***Scheme S2****.* Synthetic route of HA-PBA.

***Scheme S3****.* Synthetic route of OHA-PBA.

***Figure S1****.* ^1^H NMR spectrogram of (A) HA and (B) OHA-PBA. (D_2_O as a solvent, 300MHz NMR).

***Figure S2***. FTIR spectrogram of the polymers of HA, OHA-PBA and HA-PBA.

***Figure S3***. (A) Optical photographs after inversion for 30s after gel formation for the OPDL gels with different composition ratios, the values given in the table refer to the final concentration of OPDL gel after formation, so the concentration of precursor solution should be twice the given value. (B) Pictures of hydrogels prepared with different proportions of feed after standing for 2 hours. (C) Oxidation degree of the repeated OHA-PBA samples determined using both hydroxylamine hydrochloride titration and Purpald reagent methods. (D) Images of the OPDL gels prepared by the different samples.

***Fi*gure *S4****.* Figure S4. Images of the injectability of OPDL gel (Gel-2) with syringes of different specifications performed by different volunteers.

***Figure S5****.* Rheological performance test of OPDL gel before and after the “contact self-healing” at a fixed strain of 1%, F=1hz.

***Figure S6****.* (A) The original graph of Dex's UV spectrum standard curve, the absorbance at a wavelength of 242 nm was used to calculate the characteristic, with the standard solution solvent as PBS (0.02 M pH~7.4), (B) The degradability of OPDL gel in the presence of hyaluronidase and cell culture-grade pancreatic protease solution environments respectively.

***Figure S7****.* Images of the RBCs after treated with OPDL gel and TegadermTM, with PBS (0.02 M pH~7.4) as negataive control and T-100 (Triton X-100) as positive control, the unit of concentration was µg/mL.

***Figure S8****.* Concentration-dependent toxicity test, and the cell migration test of OPDL gel on neuralstem cells.

***Figure S9****.* Zoomed images of Figure 4D, the blue box shows typical foam-like cavities caused by inflammation-induced edema, the yellow arrows indicating mild capillary tearing in the local area. (scale bar is 100 um)

***Figure S10*.** Statistical analysis of bladder slice thickness in SD rats after SCI treatment experiment.

***Figure S11*.** Gait analysis of the experimental rats after treated for 8 weeks. The stride length, print area, step sequence and stance width was calculated respectively, for the assessment of the motor ability (n=6).

***Figure S12***. Fluorescence quantitative assay of toxic aldehydes in PC12 cells before and after treated with hydrogel samples.

***Figure S13.*** Statistics of colony formation of viable bacteria after contact with OPDL gel for different time respectively.

**Figures and schemes.**


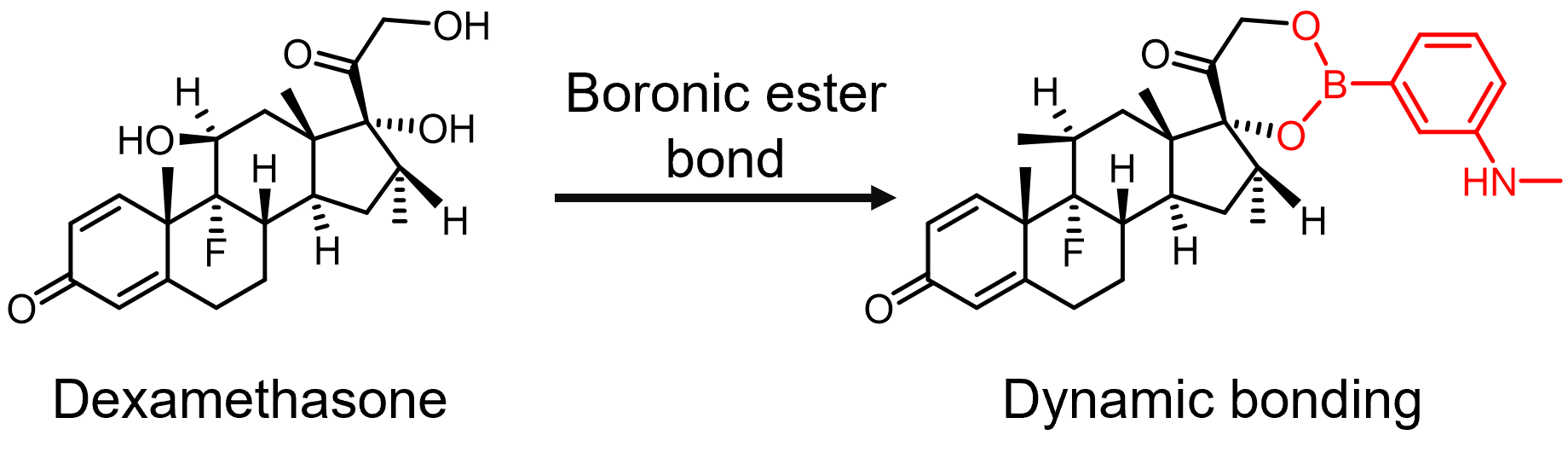


**Scheme S1**. Schematic diagram of the loading mechanism of OPDL gel for Dex molecules.


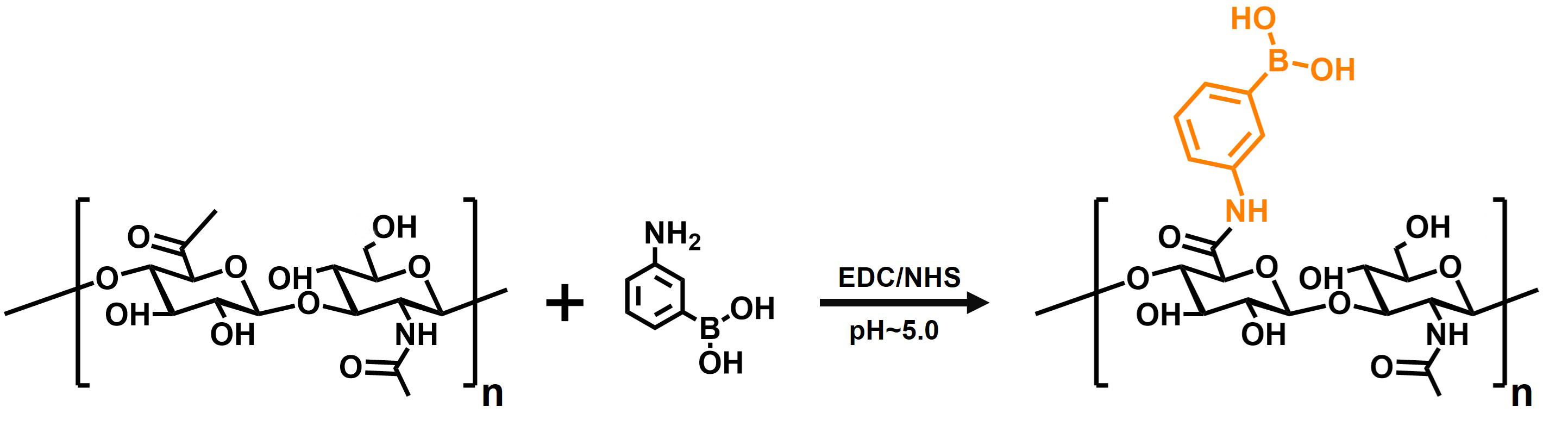


**Scheme S2**. Synthetic route of HA-PBA.


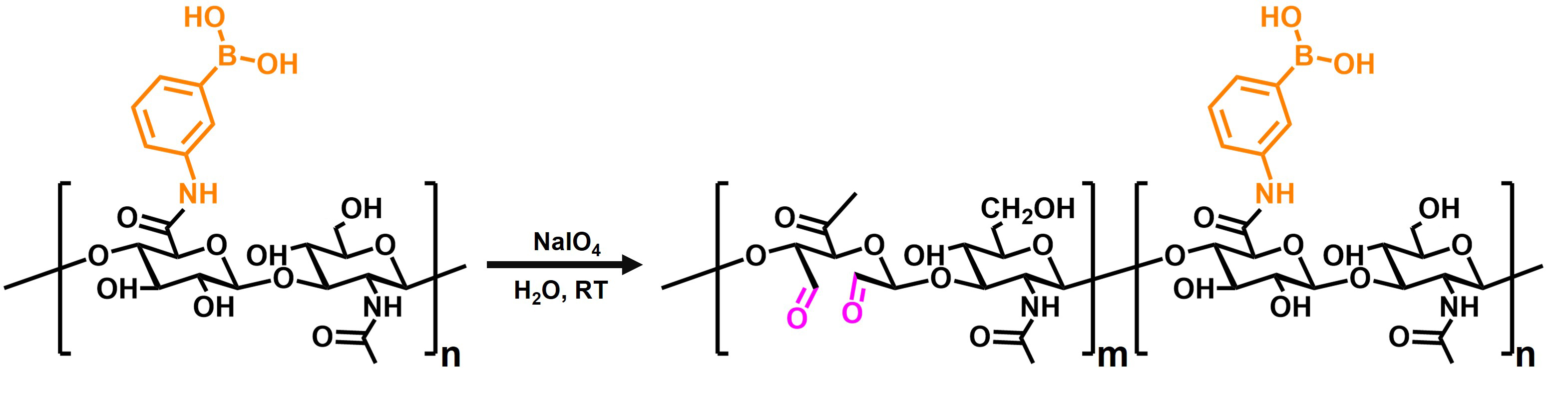


**Scheme S3**. Synthetic route of OHA-PBA.


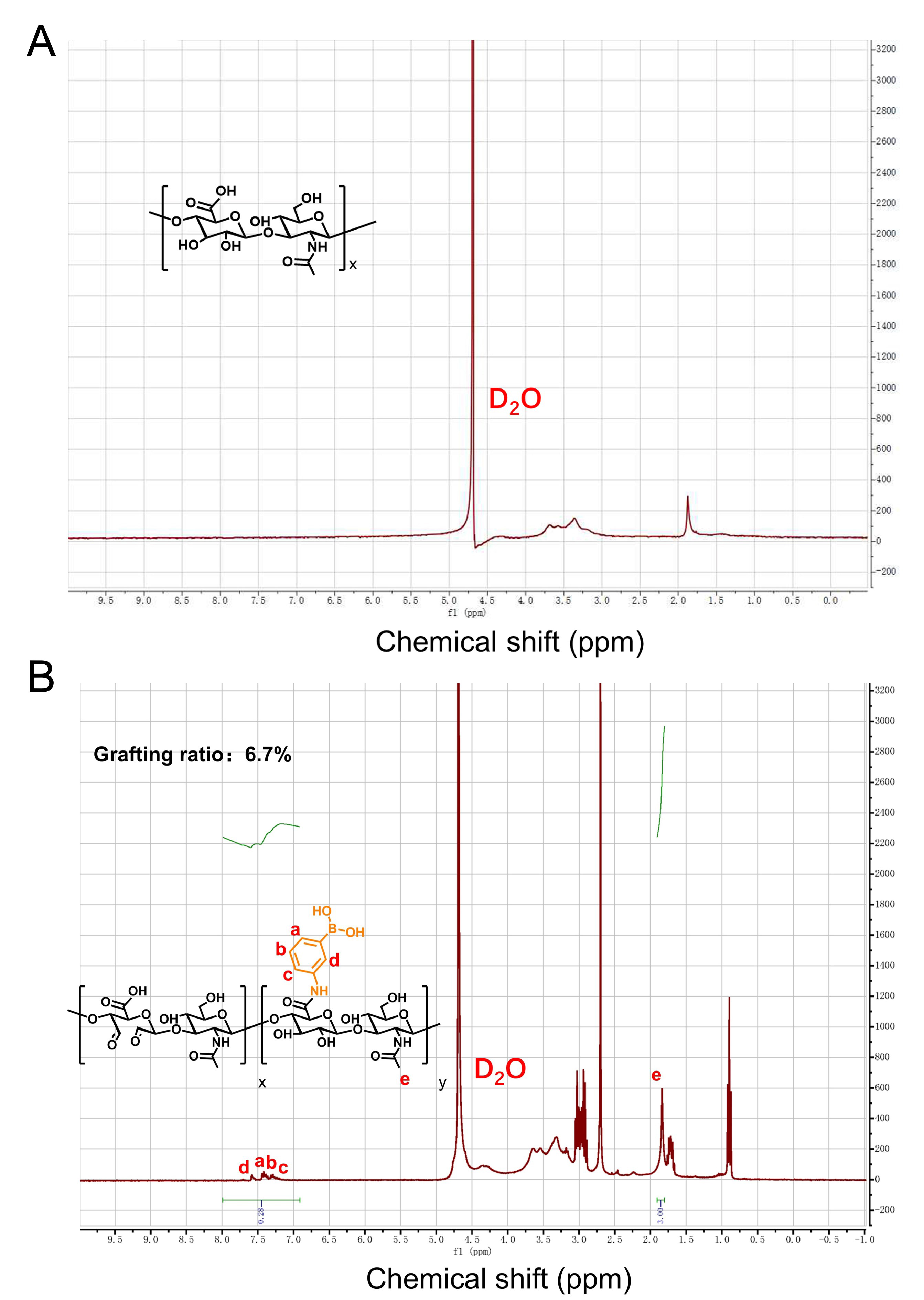


**Figure S1**. ^1^H NMR spectrogram of (A) HA and (B) OHA-PBA. (D_2_O as a solvent, 300MHz NMR).


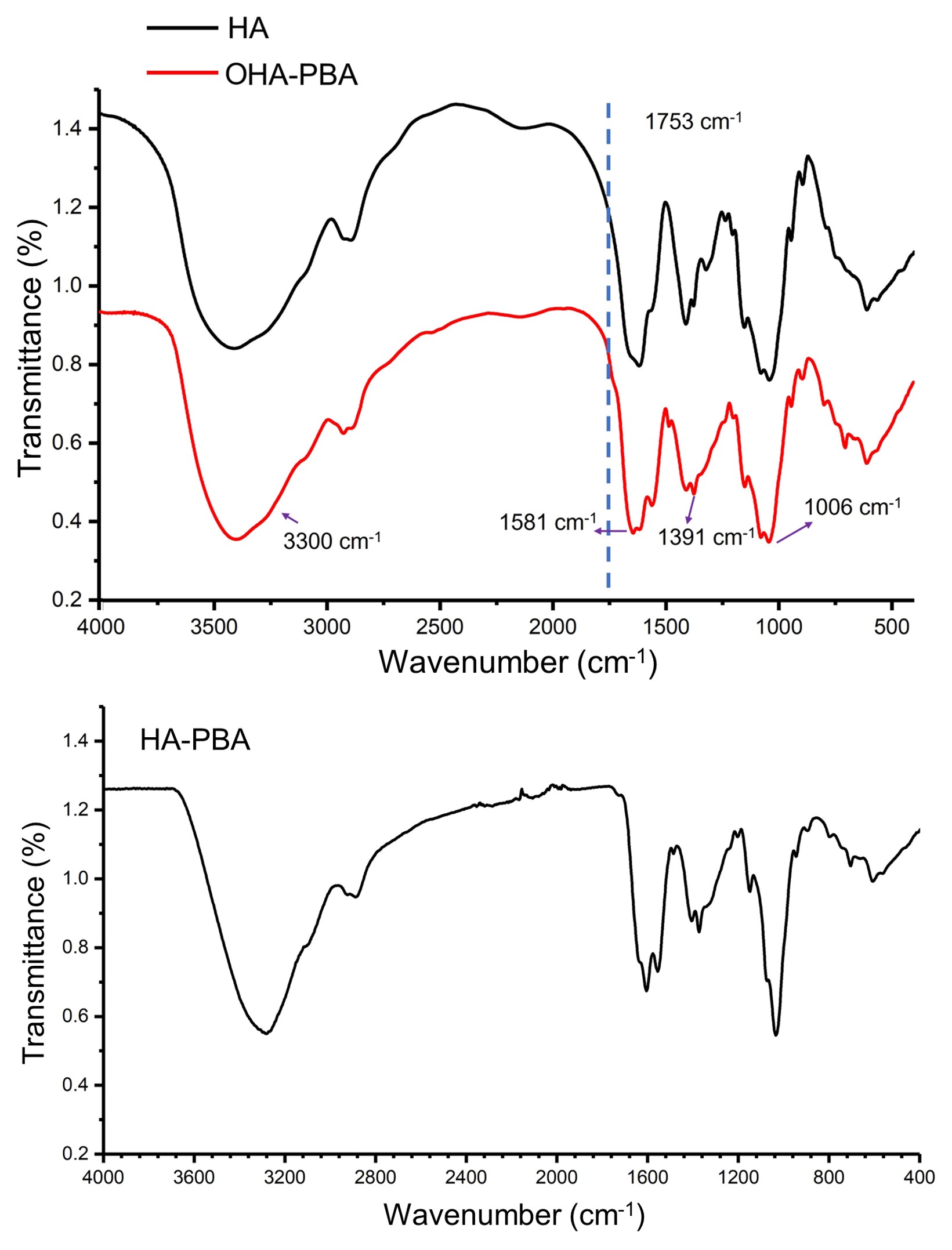


**Figure S2**. FTIR spectrogram of the polymers of HA, OHA-PBA and HA-PBA.


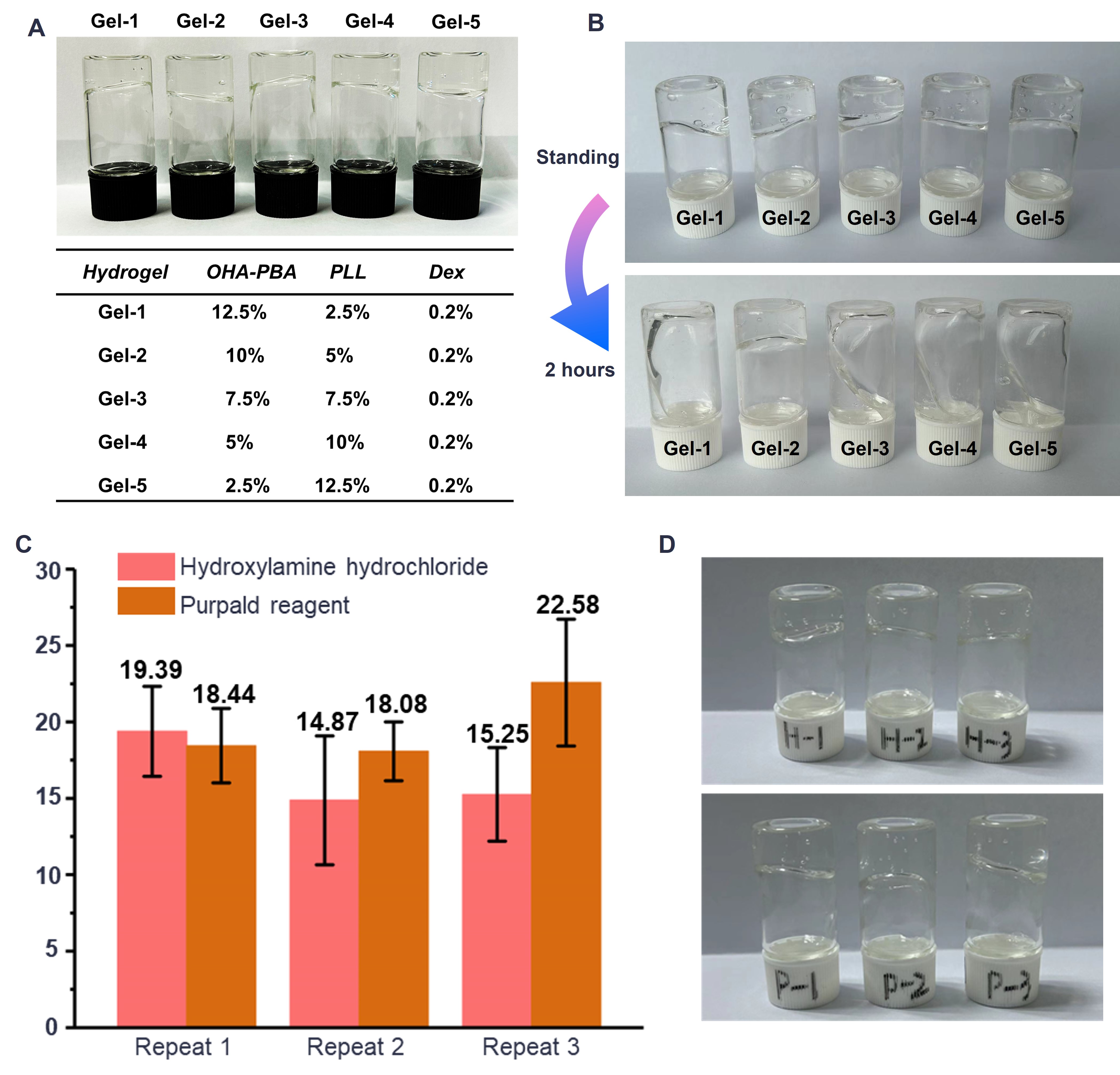
 **Figure S3**. (A) Optical photographs after inversion for 30s after gel formation for the OPDL gels with different composition ratios, the values given in the table refer to the final concentration of OPDL gel after formation, so the concentration of precursor solution should be twice the given value. (B) Pictures of hydrogels prepared with different proportions of feed after standing for 2 hours. (C) Oxidation degree of the repeated OHA-PBA samples determined using both hydroxylamine hydrochloride titration and Purpald reagent methods. (D) Images of the OPDL gels prepared by the different samples.


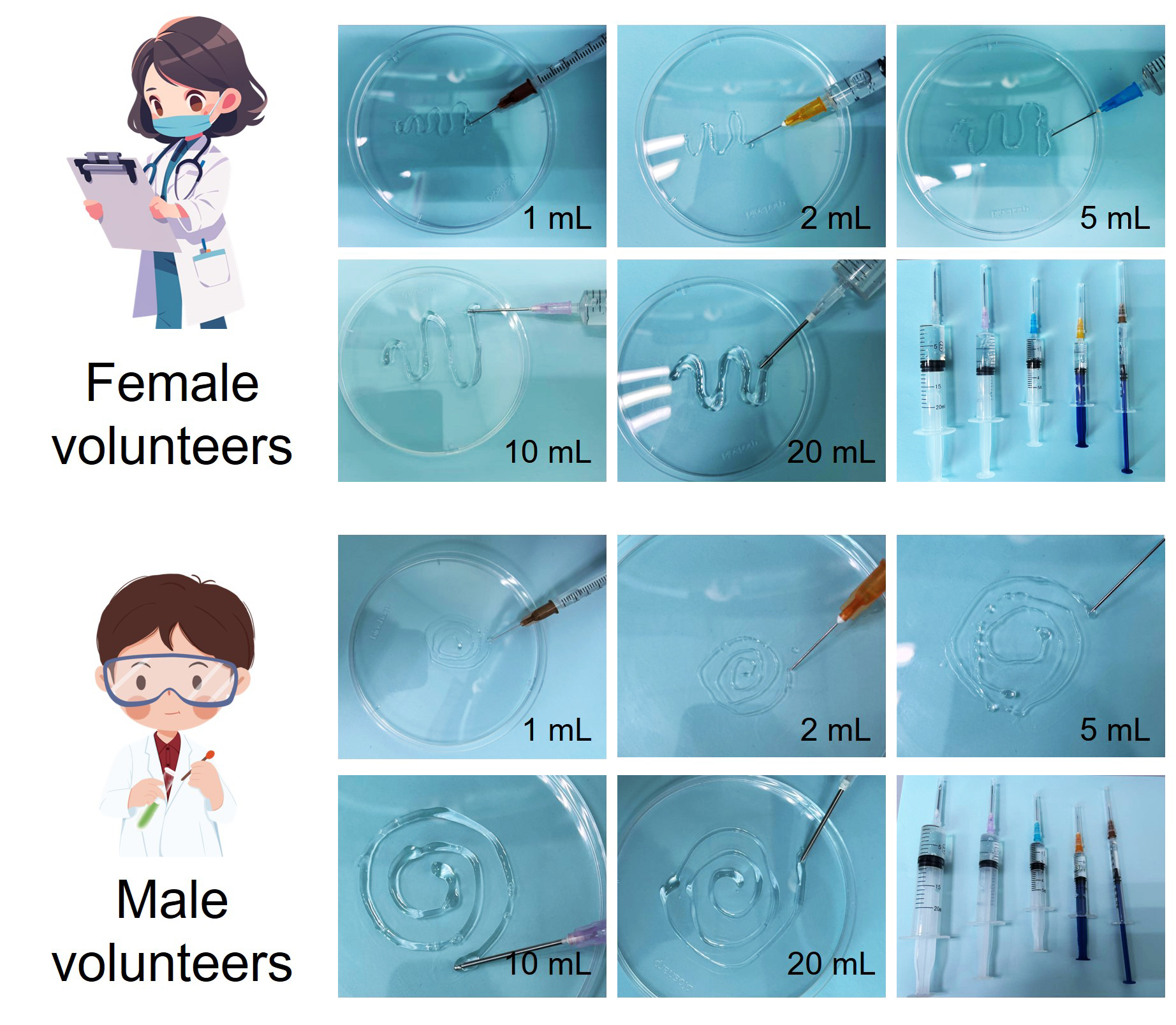


**Figure S4**. Images of the injectability of OPDL gel (Gel-2) with syringes of different specifications performed by different volunteers.


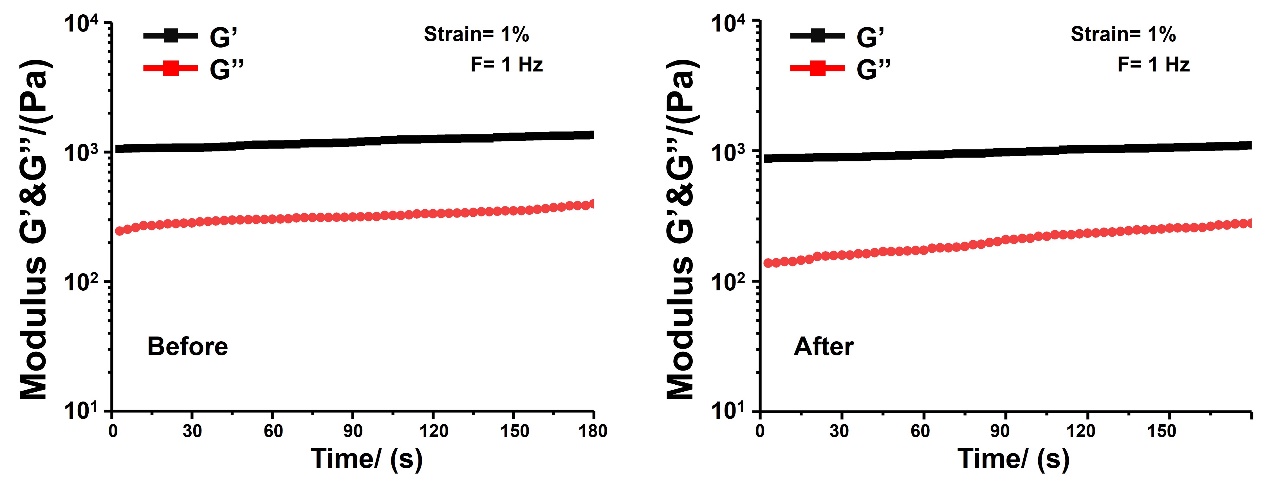


**Figure S5**. Rheological performance test of OPDL gel before and after the “contact self-healing” at a fixed strain of 1%, F=1hz.


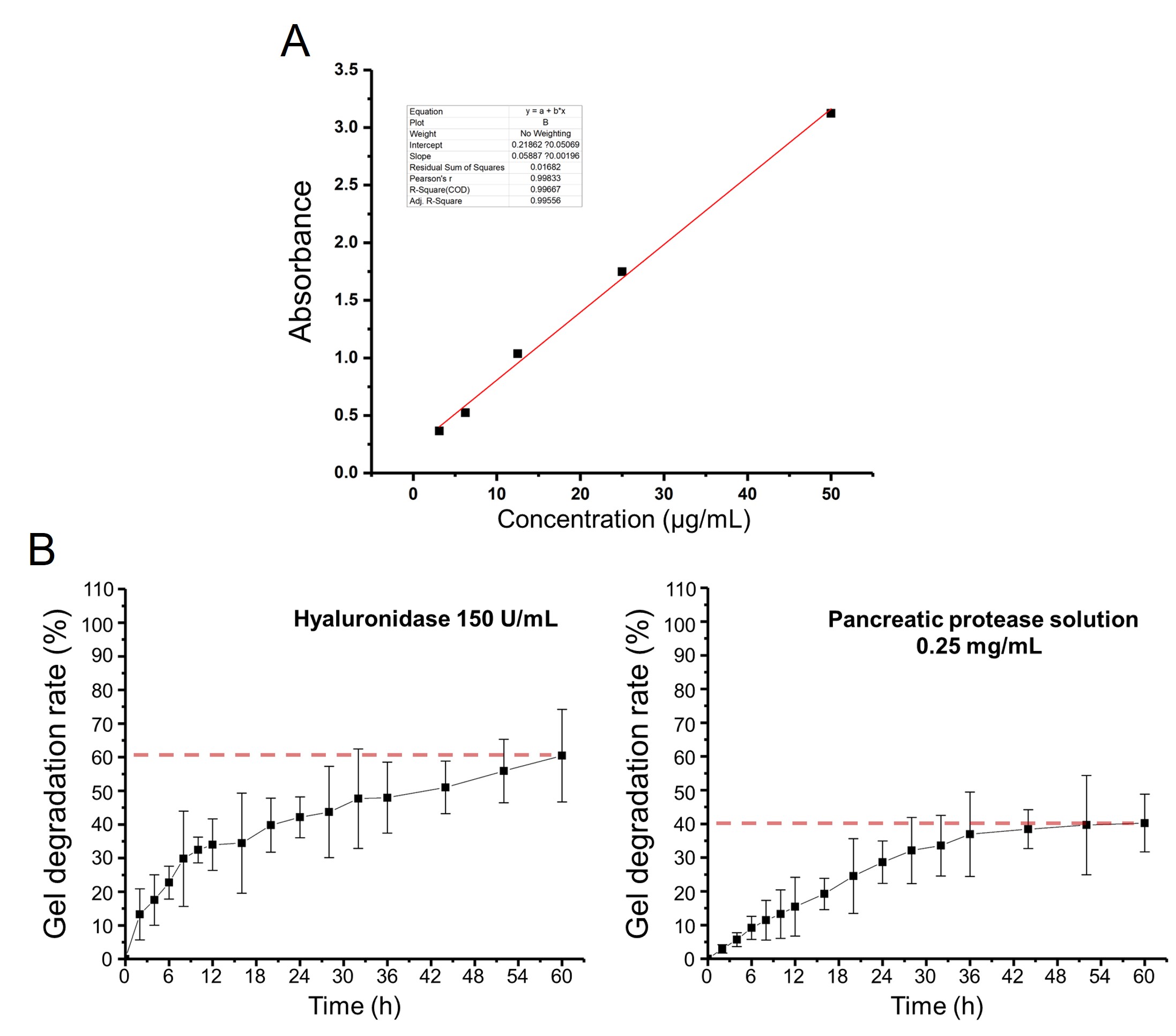


**Figure S6**. (A) The original graph of Dex's UV spectrum standard curve, the absorbance at a wavelength of 242 nm was used to calculate the characteristic, with the standard solution solvent as PBS (0.02 M pH~7.4), (B) The degradability of OPDL gel in the presence of hyaluronidase and cell culture-grade pancreatic protease solution environments respectively.


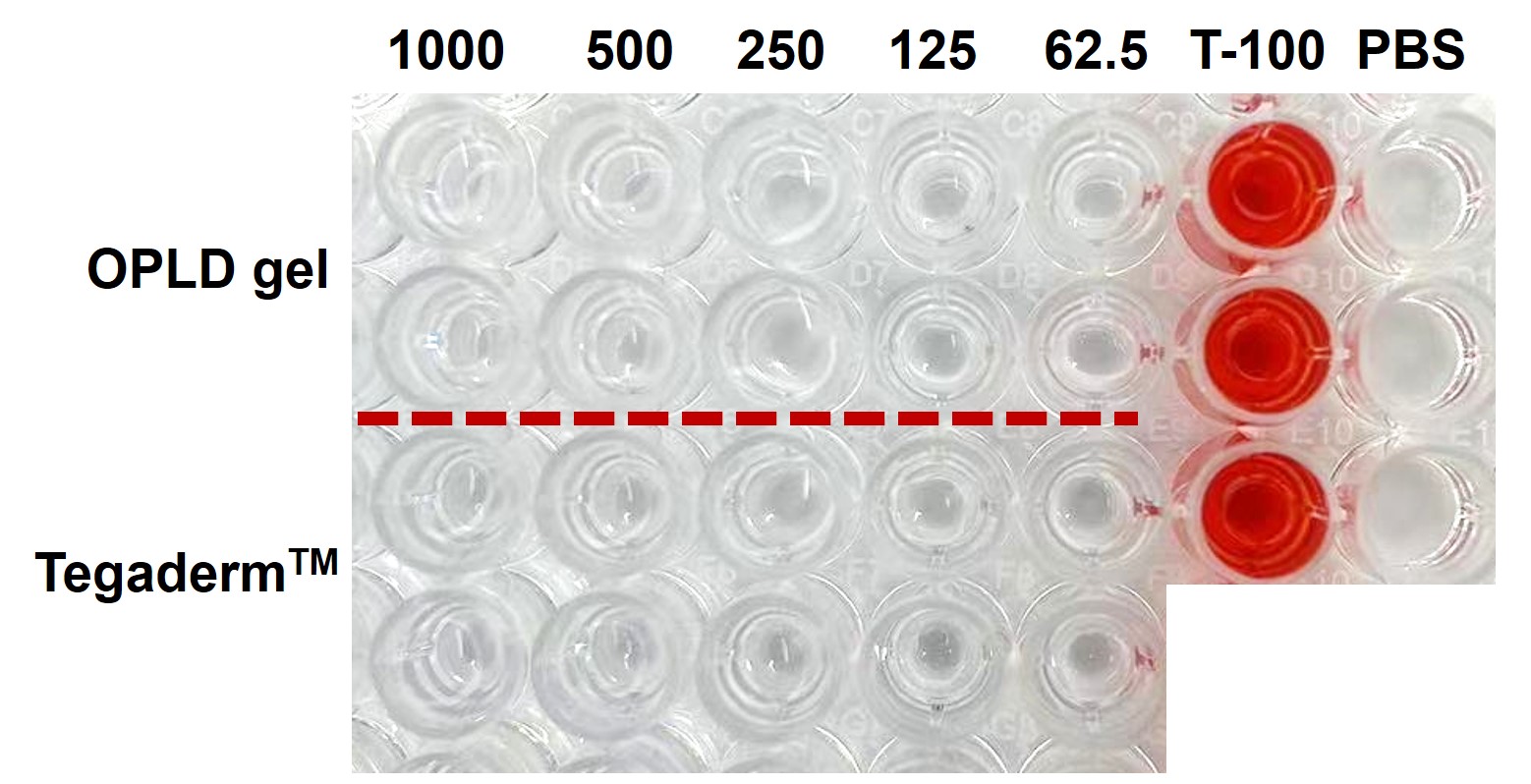


**Figure S7.** Images of the RBCs after treated with OPDL gel and Tegaderm^TM^, with PBS (0.02 M pH~7.4) as negataive control and T-100 (Triton X-100) as positive control, the unit of concentration was µg/mL.


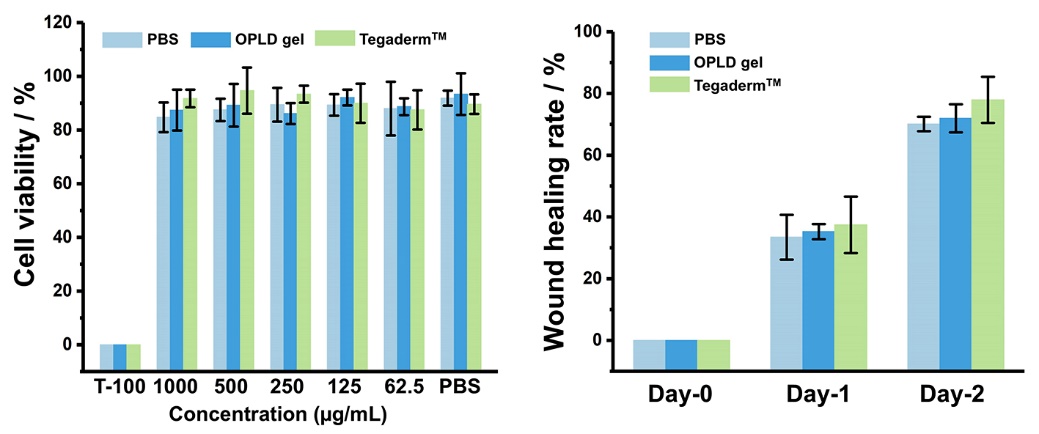


**Figure S8.** Concentration-dependent toxicity test, and the cell migration test of OPDL gel on neuralstem cells.


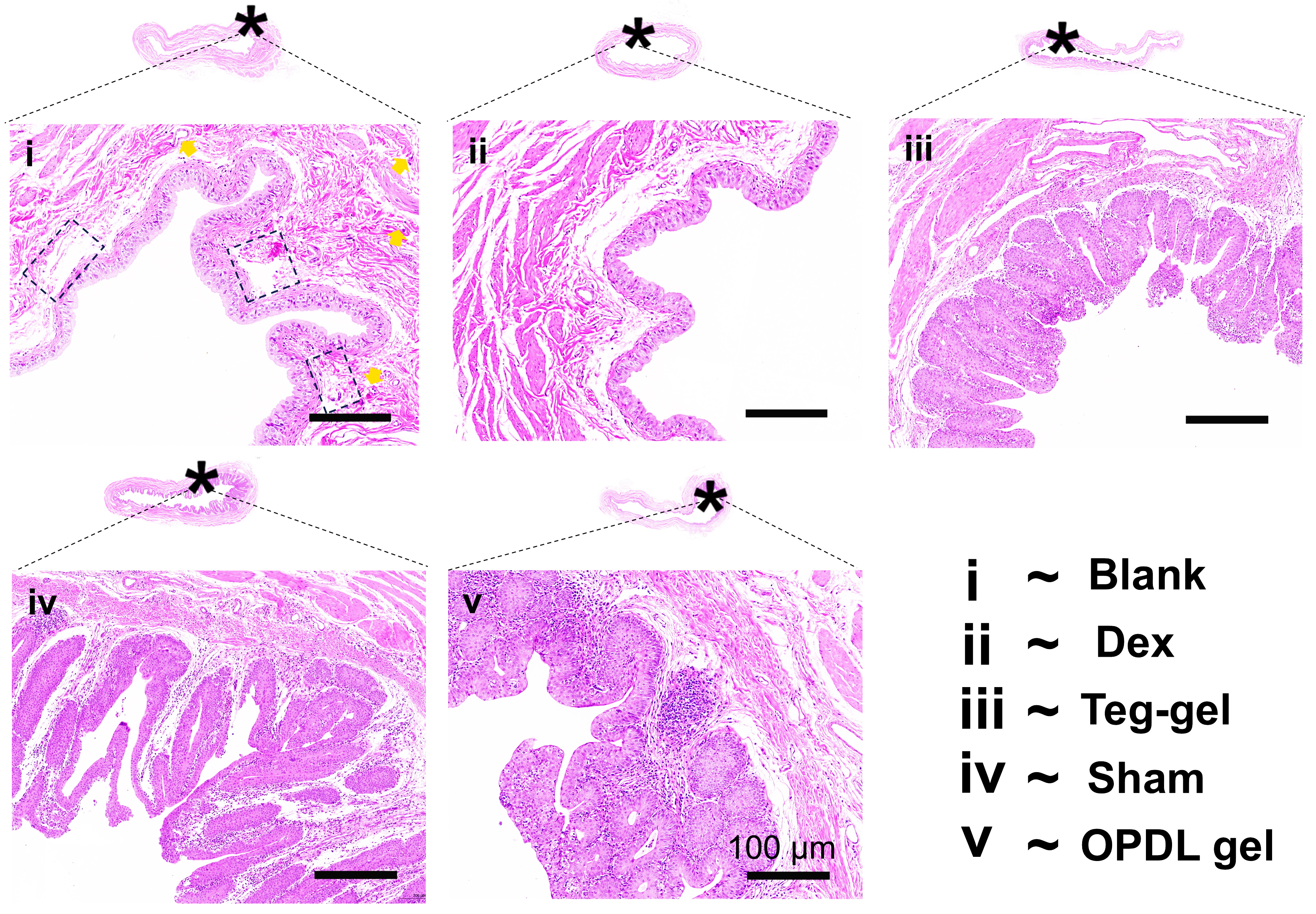


**Figure S9.** Zoomed images of Figure 4D, the blue box shows typical foam-like cavities caused by inflammation-induced edema, the yellow arrows indicating mild capillary tearing in the local area. (scale bar is 100 um)


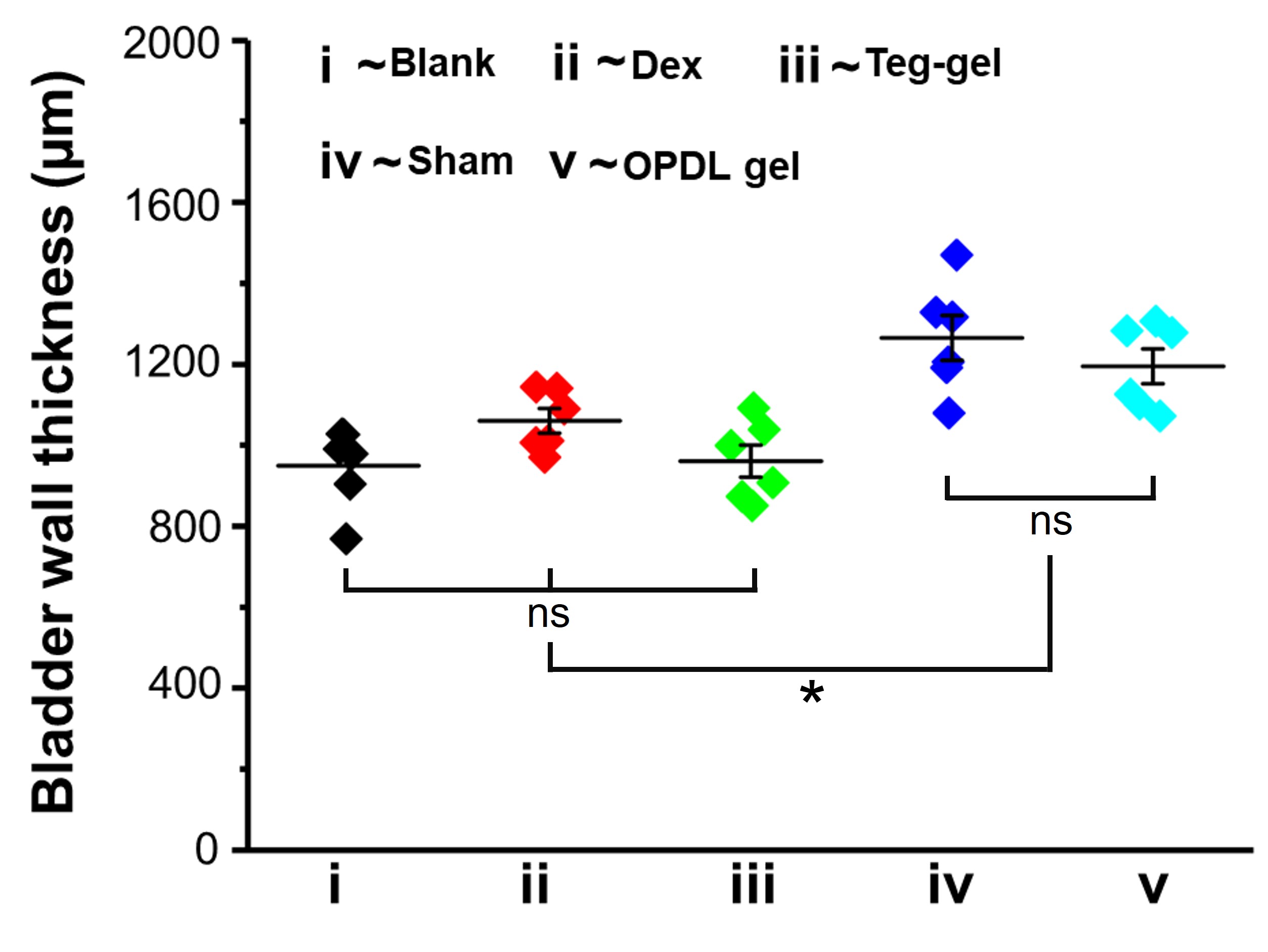


**Figure S10.** Statistical analysis of bladder slice thickness in SD rats after SCI treatment experiment.


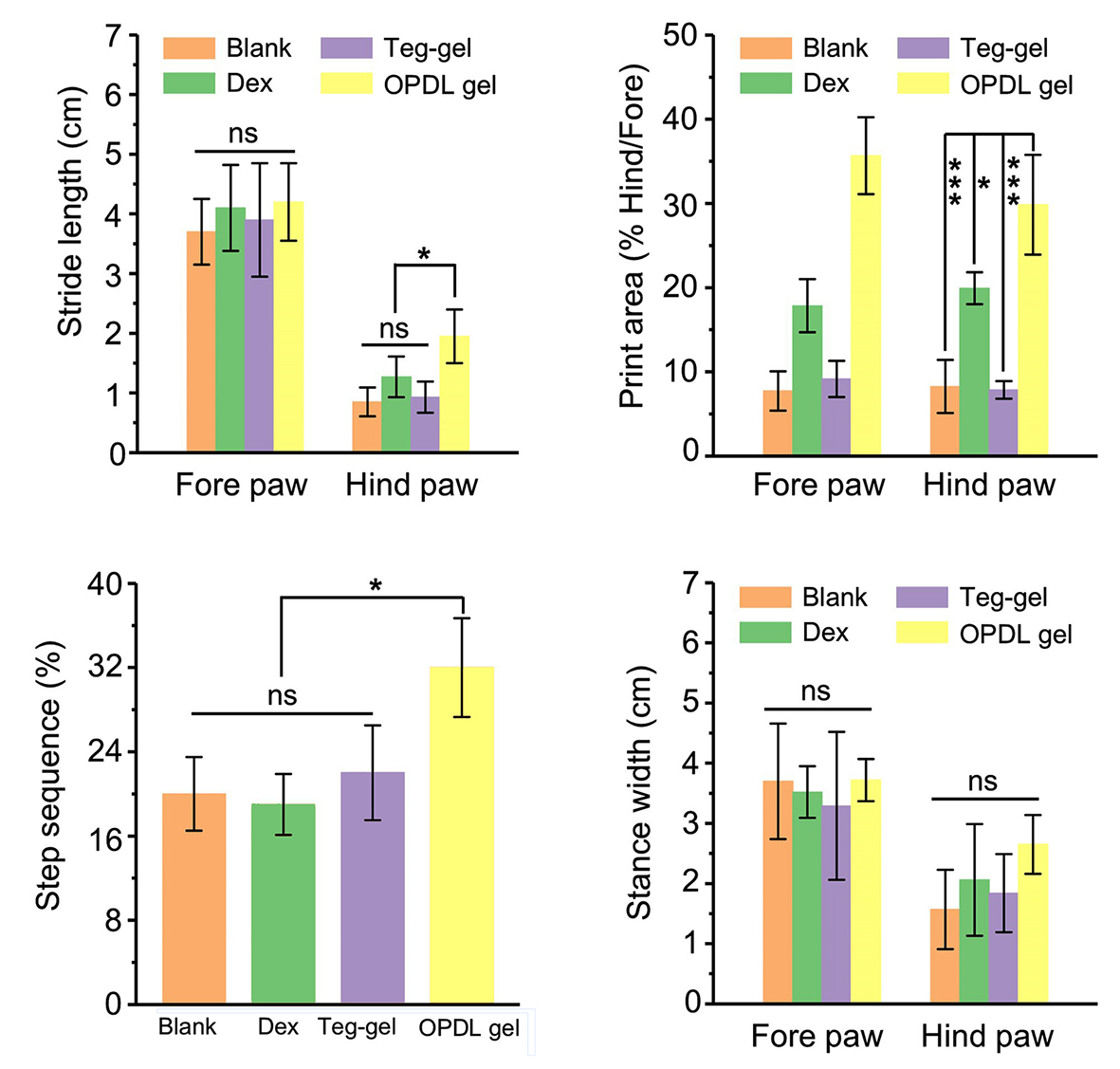


**Figure S11.** Gait analysis of the experimental rats after treated for 8 weeks. The stride length, print area, step sequence and stance width was calculated respectively, for the assessment of the motor ability (n=6).


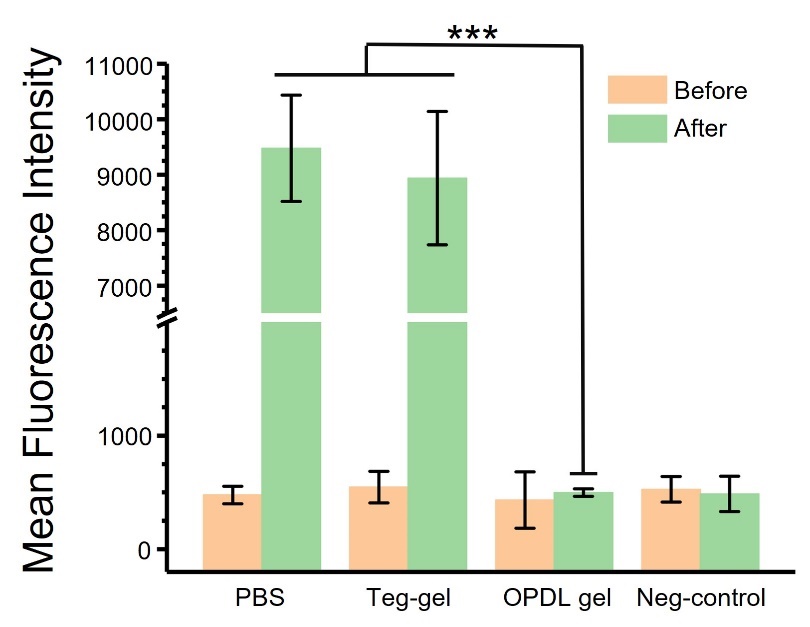


**Figure S12**. Fluorescence quantitative assay of toxic aldehydes in PC12 cells before and after treated with hydrogel samples.


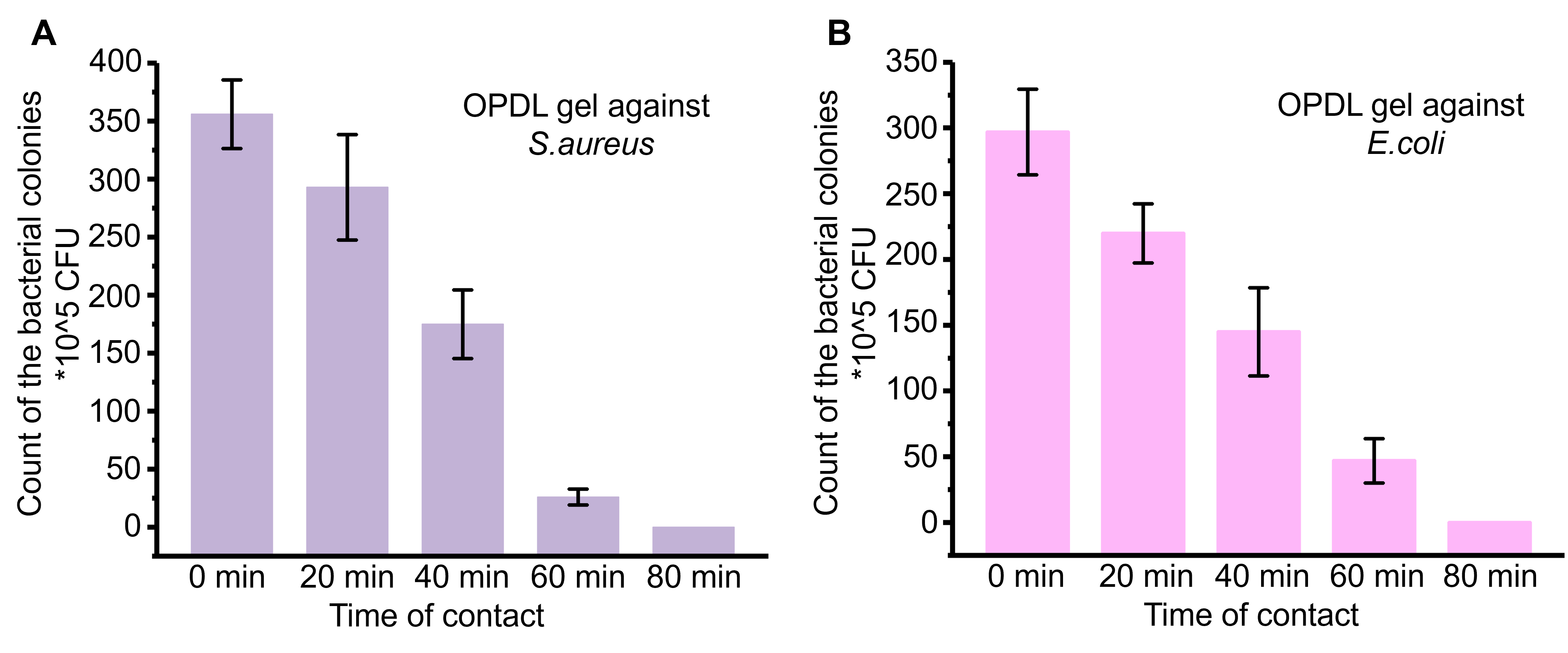


**Figure S13.** Statistics of colony formation of viable bacteria after contact with OPDL gel for different time respectively.
